# Insights into homeobox B9: a propeller for metastasis in dormant prostate cancer progenitor cells

**DOI:** 10.1038/s41416-021-01482-y

**Published:** 2021-07-10

**Authors:** Yi Sui, Wei Hu, Wei Zhang, Dejian Li, Hongbo Zhu, Qinghua You, Rujian Zhu, Qingtong Yi, Tao Tang, Lili Gao, Shengjuan Zhu, Tao Yang

**Affiliations:** 1 Department of Nutrition, The First Affiliated Hospital of Sun Yat-sen University, Guangzhou, China; 2 Department of Urology, Shanghai Pudong Hospital, Fudan University Pudong Medical Center, Shanghai, China; 3 Department of Pharmacology, School of Basic Medicine, Hebei University of Chinese Medicine, Shijiazhuang, China; 4 Department of Othopedics, Shanghai Pudong Hospital, Fudan University Pudong Medical Center, Shanghai, China; 5 Department of Pathology, Shanghai Pudong Hospital, Fudan University Pudong Medical Center, Shanghai, China; 6 Department of Obstetrics and Gynaecology, Prince of Wales Hospital, The Chinese University of Hong Kong, Hong Kong SAR, China; 7 Center for Medical Research and Innovation, Shanghai Pudong Hospital, Fudan University Pudong Medical Center, Shanghai, China

**Keywords:** Prostate cancer, Cancer stem cells

## Abstract

**Background:**

Metastasis is the major cause of treatment failure and cancer-related deaths in prostate cancer (PCa) patients. Our previous study demonstrated that a CD44^+^ subpopulation isolated from PCa cells or tumours possesses both stem cell properties and metastatic potential, serving as metastatic prostate cancer stem cells (mPCSCs) in PCa metastasis. However, the underlying mechanisms remain unknown.

**Methods:**

In this study, we established PCa models via the orthotopic and subcutaneous implantation of different human PCa cancer cell lines, and compared the metastatic efficacy, after which process function analysis of target genes was pinpointed.

**Results:**

Several novel differentially expressed genes (DEGs) between orthotopic and ectopic tumours were identified. Among them, human homeobox B9 (HOXB9) transcription factor was found to be essential for PCa metastasis, as evidenced by the diminished number of lung metastatic foci derived from orthotopic implantation with HOXB9-deficient CWR22 cells, compared with the control. In addition, HOXB9 protein expression was upregulated in PCa tissues, compared with paracancer and benign prostate hyperplasia tissues. It was also positively correlated with Gleason scores. Gain- and loss-of-function assays showed that HOXB9 altered the expression of various tumour metastasis- and cancer stem cell (CSC) growth-related genes in a transforming growth factor beta (TGFβ)-dependent manner. Moreover, HOXB9 was overexpressed in an ALDH^+^CD44^+^CXCR4^+^CD24^+^ subpopulation of PCa cells that exhibited enhanced TGFβ-dependent tumorigenic and metastatic abilities, compared with other isogenic PCa cells. This suggests that HOXB9 may contribute to PCa tumorigenesis and metastasis via TGFβ signalling. Of note, ALDH^+^CD44^+^CXCR4^+^CD24^+^-PCa cells exhibited resistance to castration and antiandrogen therapy and were present in human PCa tissues.

**Conclusion:**

Taken together, our study identified HOXB9 as a critical regulator of metastatic mPCSC behaviour. This occurs through altering the expression of a panel of CSC growth- and invasion/metastasis-related genes via TGFβ signalling. Thus, targeting HOXB9 is a potential novel therapeutic PCa treatment strategy.

## Introduction

Prostate cancer (PCa) is the third most common cancer and the sixth leading cause of cancer-related death among males worldwide [1]. PCa incidence varies throughout the world, with the highest rates in western countries and the lowest in South Asia [2]. Although patients with localised and regional PCa are curable with a 5-year survival rate of ~100% upon diagnosis, those with distant metastases have a 5-year survival of only 29% [3]. Therefore, elucidation of the mechanisms underlying PCa progression and metastasis is required to improve the disease’s treatment efficacy and prognosis.

Metastasis is a complex multistep process that involves the migration of tumour cells from the primary tumour, and the subsequent initiation of secondary tumours in distant organs [4]. It is well known that metastasis is an inefficient process, as only about 0.2% of disseminated tumour cells form metastases in distant organs [5]. Metastatic cancer stem cells (CSCs), which comprise a small subgroup of CSCs with both stem cell properties and metastatic potentials, are believed to be metastatic precursors that drive tumour invasion and metastasis [6]. There is a close association between CSC markers and cell phenotypes. CD44 [7], CD24 [8], CD133 [9], aldehyde dehydrogenase (ALDH) [10], integrin α2β1 [11] and C-X-C chemokine receptor type 4 (CXCR4) [12] are well-accepted CSCs markers in PCa. CD44^+^ PCa cells are more proliferative, clonogenic, tumorigenic, and metastatic than the isogenic CD44^−^ PCa cells [13]. A Higher CD44^+^ population was showed in Docetaxel-resistant PCa cells and promotes migration and invasion probably via induction of Hippo-Yap signialing pathway [14]. This suggests that CD44^+^ subpopulations may act as metastatic prostate CSCs (mPCSCs). CD24 is a significant predictor for PCa relapse and poor prognosis [15]. Circulating tumour cells (CTCs) from patients with metastatic castration-resistant prostate cancer (mCRPC) have robust CD133 protein expression and high levels of CD133 are associated with increased capacity for proliferation [16]. High ALDH activity can be used to identify tumour initiating and metastasis initiating cells in PCa [17]. Integrin α2β1 is needed for the efficient metastasis of PCa cells to the skeleton [18]. PCa and perhaps other neoplasms (e.g., breast) may use the chemokine stromal cell-derived factor-1 (SDF-1 or CXCL1) and its receptor CXCR4 to promote their spreading to the bone and other tissues [19, 20]. Similarly, CD133^+^CXCR4^+^ cells, but not CD133^+^CXCR4^−^ cells, are essential for pancreatic tumour metastasis [21]. Furthermore, CD44^+^CD24^−/low^ breast cancer CSCs isolated from both primary tumours and lung metastatic foci can generate orthotopic primary tumours and subsequent lung metastases [22]. This provides the most direct evidence of the presence of metastatic CSCs and for the CSCs’ contribution to metastasis. Although the link between genetic signatures and CSCs’ metastatic potential has been established, the regulatory mechanisms remain unclear.

It has been established that the orthotopic implantation of cancer in nude mice produces distant metastases, whereas subcutaneous transplantation does not [23, 24]. This indicates the importance of tumour microenvironments in metastasis. The differential gene expression profiles of orthotopic and ectopic tumour implantation models may provide clues to understanding metastatic mechanisms. In this study, we compare the metastatic rates of tumour models established via orthotopic and subcutaneous implantation using different human PCa cell lines. To do so, PCa cell lines with the highest metastatic rate were selected for further analysis of the differentially expressed genes (DEGs) in orthotopic and ectopic tumours. The findings show that human homeobox B9 (HOXB9), a key transcription factor that promotes metastases in lung and breast cancers [25, 26], is involved in enhanced metastatic capacity and resistance to physical/chemical castration therapy in the PCa model caused by orthotopic implantation of ALDH^+^ CD44^+^ CXCR4^+^ CD24^+^ subpopulation. Our study reveals that HOXB9 can regulate the expression of a panel of CSC growth- and invasion/metastasis-related genes in mPCSCs via TGFβ signalling. This suggests that HOXB9 is a novel therapeutic target in PCa treatment.

## Materials and methods

### Patients and tissue samples

Para-carcinoma and PCa tissues were obtained from 32 patients with primary PCa who had undergone radical prostatectomy at the Department of Urology, Fudan University Pudong Medical Center, between January 1998 and July 2018, for patient-derived xenograft (PDX) study, as well as tumour cell phenotyping and in vivo inoculation assay. Benign prostate hyperplasia tissues were obtained from diagnostic prostate needle biopsies. All tissue samples were processed immediately after surgical removal. Diagnosis and grading were histologically confirmed by two experienced pathologists, according to the Gleason grading system. The tumour tissues were fixed in 10% formalin, embedded in paraffin and cut into 4–7-μm thick sections. For correlation analysis in terms of HOXB9 expression level, Gleason scores and prognostic features, patients data and tissue samples (*n* = 12; starting from January 1998 until October 2015) were retrieved from the Department of Health Information in Fudan University Pudong Medical Center. This study was approved by the Ethics Committee of Fudan University Pudong Medical Center, as well as local authorities. Written consent was obtained from all patients.

### Animals

Six- to eight-week-old male NOD/SCID mice weighing 22–25 g were produced from our own breeding colonies or purchased from Jackson Laboratories (Bar Harbor, ME, USA). They were maintained in standard conditions according to institutional guidelines. All animal care and procedures described in this study were approved by the Ethics Committee of Shanghai Pudong Hospital (Shanghai, China). All animal experiments were performed in accordance with the guidelines for the proper conduct of animal experiments, as stipulated by Shanghai Pudong Hospital and Fudan University (Shanghai, China).

### Cell culture

PCa cells (CWR22, Du-145, LNCaP, LAPC4 or LAPC9) were obtained from American Type Culture Collection (ATCC, Manassas, VA, USA). CWR22, Du-145 and LNCaP were cultured in RPMI-1640 plus 8% heat-inactivated foetal bovine serum (FBS); whereas LAPC4 and LAPC9 were maintained in NOD/SCID male mice. CWR22, Du-145 or LNCaP cells overexpressing GFP or RFP, and CWR22 cells overexpressing luciferase (Luc-CWR22) were cultured, as previously described [27, 28], and maintained in RPMI-1640 supplemented with 8% FBS at 37 °C in a humidified atmosphere of 5% CO_2_.

### Tumour models comparing orthotopic and ectopic implantation

Mice were randomly separated into different groups (*n* = 12/group). Animals were anaesthetised with inhalation of 3% halothane and maintained on 1.5% halothane in 70% nitrous oxide and 30% oxygen. In total, 1 × 10^5^ PCa cells (CWR22, Du-145, LNCaP, LAPC4 or LAPC9) were injected into the dorsal lobe of the prostate or subcutaneous area of the same mouse. After that, mice were placed at 37 °C and monitored in micro-isolator cages (one per cage) until they recovered from surgery. Mice were sacrificed by CO_2_ asphyxiation at 2 months after the initial appearance of tumours, and the primary tumours were immediately harvested, weighed, and stored at –80 °C until use. GFP- or RFP-expressing primary tumours and metastases in miscellaneous organs (bilateral lungs and kidneys, pancreas, liver, spleen, brain and bone marrow) were visualised, and representative images were acquired using Nikon SMZ1500 whole-mount epifluorescence dissecting microscopy (Nikon, Japan). The metastasis rate was calculated as the number of metastatic foci detected in each group/(the number of detected organs × the number of mice in each group).

### Microarray analysis

The gene expression profile of each sample was examined using an Affymetrics Genechip (ThermoFisher Scientific, Waltham, MA, USA) containing probes covering 8500 protein-coding genes following the manufacturer’s standard protocols. The DEGs between each group were screened using a random variance model (RVM) *t* test. Genes for expression levels with *P* value <0.05 and fold change >2 were considered to be DEGs. Cluster analysis was performed using hierarchical clustering.

### Immunofluorescence staining

The tissue sections were subject to HOXB9 immunostaining according to the standard protocol [29]. Sections were incubated with mouse anti-human HOXB9 antibody (Millipore, Billerica, MA, USA). Sections were further incubated with tetramethylrhodamine isothiocyanate (TRITC)-conjugated goat anti-mouse IgG (1:100, Millipore). Rinsed sections were counterstained with 10 µg/ml 4’,6-diamidino-2-phenylindole (DAPI, Sigma, St. Louis, MO, USA). Normal IgG was used as a negative control. An inverted fluorescence microscope (IX83, Olympus, Tokyo, Japan) was used for visualisation, and red staining was considered HOXB9-positive.

### CSC growth-related DEG knockdown in CWR22-GFP cells and the establishment of an orthotopic tumour model

The vectors expressing shRNAs of 12 CSC growth-related genes (Supplementary Table 1), such as CXCR4 (C-X-C chemokine receptor type 4), CD133 (Prominin-1), ABCG2 (ATP-binding cassette subfamily G member 2), CD24 (signal transducer CD24), HOXB9 (homeobox protein Hox-B9), NOS2A (inducible nitric oxide synthase), TROP2 (tumour-associated calcium signal transducer 2), LRIG1 (leucine-rich repeats and immunoglobulin-like domains protein 1), WNT4 (wingless-related integration protein 4), ID3 (DNA-binding protein inhibitor ID3), NKX3.1 (NK-3 transcription factor, locus 1) and SMAD1 (mothers against decapentaplegic homologue 1), were obtained from ThermoFisher Scientific. A control shRNA plasmid that encodes of a scrambled shRNA sequence was obtained from Santa Cruz Biotechnology (Santa Cruz, CA, USA). CWR22-GFP cells were transfected with control shRNA or each shRNA using pGC-silencer-U6/Neo/GFP and selected using G418 to generate respective stable cell lines. In total, 1 × 10^4^ stable cells with deficiencies of each gene were implanted into the dorsal lobe of each mouse’s prostate (*n* = 12). In NOD-SCID male mice supplemented with dihydrotestosterone pellets (0.2 mg/mouse embedded subcutaneously), the GFP^+^-metastatic foci in the lungs were visualised and counted at week 12 post-transplant with a Nikon SMZ1500 microscope.

### Flow cytometry cell sorting and the establishment of orthotopic tumour models

Orthotopic Luc-CWR22 tumours were harvested at 2 weeks after implantation in mouse dorsal prostates. Single-cell suspension was prepared in phosphate-buffered saline (PBS) and incubated with FITC, APC, PE or PE-cy7-conjugated primary antibodies against ALDH (Stem Cell Technologies, Vancouver, Canada), CD44 (BD Biosciences, Bedford, MA, USA), CXCR4 (BD Biosciences), CD24 (BD Biosciences) or α2β1 (Millipore, Billerica, MA, USA), either individually or in combination. Flow cytometer (BD FACSAria III, San Jose, CA, USA) was used to separate CD44^+^-, CD44^+^α2β1^+^-, ALDH^+^CD44^+^α2β1^+^- and ALDH^+^CD44^+^CXCR4^+^CD24^+^ cell compartments. To purify ALDH^+^CD44^+^CXCR4^+^CD24^+^ cells from xenograft tumours, we incubated discrete cells with a FcR blocking agent (Miltenyi Biotec, San Diego, CA, USA) for 15 min at 4 °C. We then stained them with primary antibody to CXCR4 (cat# 566282, BD Biosciences) for 30 min on ice, followed by staining with APC-conjugated goat anti-mouse IgG (cat# 550826, BD Biosciences) for 15 min on ice. Cells were then washed three times and stained with PE-conjugated anti-CD44 antibody (cat# 550989, BD Bioscience), followed by biotinylated anti-mouse H2-Kd (cat# 130-107-891, Miltenyi Biotec) for 20 min with the aim to remove mouse-origin cells. Next, purified cells were stained with PE-cy7-conjugated anti-CD24 (cat# 561646, BD Biosciences), for 20 min. After washing with PBS, cells were incubated in a solution containing 1% bovine serum albumin (BSA) and 2.5 μg/ml insulin (I-6634, Sigma). Then, the cells were suspended in an ALDEFLUOR assay buffer (ALDEFLUOR kit, Stem Cell Technologies) containing ALDH substrate (1 μM per 1 × 10^6^ cells). They were then incubated for 40 min at 37 °C and sorted by fluorescence-activated cell sorting (FACS). Additional purification steps via BV711-conjugated anti-CD31 (cat# 745436, BD Biosciences), PE-Cy^TM^5-conjugated anti-CD3 (cat# 555341, BD Biosciences) and PE-CF594-conjugated anti-CD14 (cat# 562334, BD Biosciences) were applied to derived cells to deplete human endothelial cells, lymphocytes and monocytes, respectively. Each subset of cells (1 × 10^3^) was resuspended in a mixture of 20 μl medium and 20 μl Matrigel, with or without TGFβ inhibitor SD208 (5 μM; Millipore). After 2 h of incubation at room temperature, cells were inoculated into mouse dorsal prostates with subcutaneous implantation of 0.2 mg dihydrotestosterone into each mouse. Mice were sacrificed 120 days after implantation, and the primary tumours were harvested immediately. Luc^+^metastatic foci in nine organs (bilateral lungs and kidneys, pancreas, liver, spleen, brain and bone marrow) were visualised, and representative images were acquired using a Nikon SMZ1500 microscope (Nikon, Japan).

### Patient-derived xenograft (PDX) models

Human PCa samples were obtained after radical prostatectomy, with the written informed consent from the patients in accordance with national and institutional guidelines and with the approval of the Ethics Committee of Shanghai Pudong Hospital and the Ethical Commission of Fudan University. Gleason grade was determined by two officially certified pathologists. PCa tissues with a Gleason score of 8 were minced into around 0.5 mm^3^ pieces, followed by PBS wash twice. Each piece was then embedded subcutaneously into the male NOD/SCID mouse, with the dihydrotestosterone pellet at 0.2 mg per mouse embedded subcutaneously on the back (*n* = 7). When the xenograft tumour became palpable, it was harvested, and ALDH^+^CD44^+^CXCR4^+^CD24^+^ and ALDH^–^CD44^–^CXCR4^–^CD24^–^ cells were isolated from xenograft tumours using FACS, respectively, as described above.

### HOXB9 overexpression

In total, 1 × 10^6^ Du-145-GFP cells were collected, washed with PBS and resuspended in Nucleofector Kit V buffer (Amaxa Biosystems, San Francisco, CA, USA). In all, 3 μg of *myc*-coupled HOXB9-expressing vector (Amaxa Biosystems) was transfected into the cells using a Nucleofector device (Amaxa Biosystems), following the manufacturer’s instructions. After 48 h of transfection, G418 was added to select and maintain the stable cells.

### HOXB9 and CD44 knockdown

The vectors expressing *HOXB9* shRNA (5′-CCC TTC AAT TTG TAG ACT CTT-3′ and 5′-CTC CTC AAT CTG AGT GAG AGA-3′; ThermoFisher Scientific) and CD44 (5′-GAC CTC TGC AAG GCT TTC AAT-3′ and 5′-ATT GAA AGC CTT GCA GAG GTC-3′; Santa Cruz Biotechnology) were transduced into Du-145 cells using FuGENE 6 (Roche Applied Science, Indianapolis, IN, USA) and Lipofectamine 2000 (Invitrogen, Carlsbad, CA, USA), respectively. Stable cells were selected and maintained using RPMI-1640 supplemented with 8% FBS.

### Semi-quantitative and quantitative RT-PCR

Total RNAs were isolated from cells or tumour tissues using Trizol (Invitrogen), and reversely transcribed to produce cDNA using a RNeasy Extraction Kit (Qiagen, Valencia, CA, USA) according to the manufacturer’s instructions. The primers for PCR are shown in Supplementary Table 2. For semi-quantitative RT-PCR, the following cycling conditions were performed: initial denaturation at 95 °C for 4 min, 40 cycles of 45 s at 94 °C, 45 s at 60 °C and 30 s at 72 °C; final extension at 72 °C for 5 min. The PCR products were analysed on 1.5% agarose gel. Images were imported with Image Lab (Bio-Rad, Hercules, CA, USA). The quantitative real-time PCR was performed in an ABI Prism 7000 Sequence Detector (Applied Biosystems, Foster City, CA, USA) using SYBR Green PCR Master Mix reagent as the detector, according to the manufacturer’s instructions. Primer sequences were as follows: *NanogP8* (forward) CGC CCT GCC TAG AAA AGA CAT TT, (reverse) ACG AGT TTG GAT ATC TTT AGG GTT TAG AAT C; β-actin (forward) CGC ACC ACT GGC ATT GTC AT, (reverse) TTC TCC TTG ATG TCA CGC AC. The target gene expression levels were normalised to the β-actin level using the comparative C_T_ method. Data are presented as fold changes in expression relative to control.

### Western blot analysis

Cell or tumour tissue lysates were collected and quantified following standard protocols. In total, 20 μg of protein samples were separated by 12% sodium dodecyl sulfate–polyacrylamide gel electrophoresis, transferred to a nitrocellulose membrane, blocked for 1.5 h with Tris-buffered saline containing Tween 20 (TBST) containing 1% BSA at room temperature, and incubated overnight with primary HOXB9 antibodies (1:1000, mouse mAb, cat# MA519117, clone 2E8, Invitrogen), TGFβ2 (1:1000, mouse mAb, cat# MAB612100, clone 8607, R&D Systems, Minneapolis, MN, USA); MMP9 (1:1000, rabbit mAb, cat# ab137867, clone EP1255Y, Abcam, Cambridge, MA, USA), CD133 (1:500, mouse mAb, cat# MAB4399-I, clone 17A6.1, Millipore), SPP1 (1:1000, goat pAb, cat# AF1433, R&D Systems), Smad1 (1:1000, mouse mAb, cat# 05-1459, clone AS22, Millipore), phospho-Smad1 (p-Smad1, 1:500, rabbit pAb, cat# 06-702, Millipore), Smad2 (1:1000, rabbit mAb, cat# 5339, clone D43B4, Cell Signaling Technology, Beverly, MA, USA), phospho-Smad2 (p-Smad2, 1:500, rabbit mAb, cat# 18338, clone E8F3R, Cell Signaling Technology), Nanog (1:1000, mouse mAb, cat# MABD24, clone 7F7.1, Millipore), Octamer-4 (OCT4, 1:1000, mouse mAb, cat# MAB4419, clone 7F9.2, Millipore), Sox2 (1:1000, mouse mAb, cat# MAB4423, clone 10H9.1, Millipore), FoxD3 (1:1000, rabbit mAb, cat# 2019, clone D20A9, Cell Signaling Technology), ABCG2 (1:500, rabbit mAb, cat# ab207732, clone EPR20080, Abcam), aldehyde dehydrogenase (ALDH, 1:1000, rabbit pAb, cat# ABD12, Millipore), CXCR4 (1:1000, rabbit mAb, cat# ab124824, clone UMB2, Abcam), PSA (1:1000, rabbit mAb, cat# 5365S, clone D6B1, Cell Signaling Technology), CD44 (1:1000, mouse mAb, cat# MA5-15462, clone 8E2F3, Invitrogen), CD24 (1:500, mouse mAb, cat# ab179821, clone EPR3006N, Abcam), prostatic acidic phosphatase (PAP, mAb, cat# MABN318, clone 3G10.1, Millipore), prostate-specific membrane antigen (PSMA, 1:1000, mAb, cat# 12815S, clone D7I8E, Cell Signaling Technology), epithelial cadherin (E-cad, 1:1000, rabbit pAb, cat# 07-697, Millipore), Slug (1:1000, rabbit mAb, cat# 9585T, clone C19G7, Cell Signaling Technology), Vimentin (1:1000, mouse mAb, cat# MABT121, clone 3CB2, Millipore), GAPDH (1:1000, rabbit mAb, cat# 2118S, clone 14C10, Millipore) and β-actin (1:2000, rabbit mAb, cat# MABT523, clone RM112, Millipore) at 4 °C. The membranes were washed three times with TBST, then incubated with horseradish peroxidase-conjugated goat anti-rabbit or anti-mouse IgG or IgM (1:2000, Millipore) for 1 h at room temperature, and washed with TBST. The chemiluminescence signal was detected using ECL (Clinx Science Instruments, Shanghai, China), and developed on X-ray film. β-actin was used as an internal control.

### Wound-healing assay

ALDH^+^CD44^+^CXCR4^+^CD24^+^ cells were seeded into a 24-well plate at a density of 5 × 10^4^ cells per well. They were then incubated either with or without 5 μM TGFβ inhibitor SD208 in 600 μl DMEM containing 0.1% FBS at 37 °C for 24 h. A cell scraper was used to generate a 2 mm-wide scratch line in the cell monolayer. Cells were allowed to migrate for 24 h and were stained with crystal violet. Cell counting was performed in ten randomly selected fields and images were captured using an inverted light microscope (IX71, Olympus).

### Transwell invasion assay

A cell invasion assay was performed using 24-well BD biocoat Matrigel invasion chambers with 8.0-µm pores (BD Bioscience Discovery Labware, Bedford, MA, USA), according to the manufacturer’s instructions. In total, 5 × 10^4^ ALDH^+^CD44^+^CXCR4^+^CD24^+^ cells were loaded into a Matrigel (100 μg/ml; BD Biosciences; diluted at 1:20 with DMEM)-coated upper chamber filled with 500 μL DMEM containing 0.1% FBS, either with or without 5 μM TGFβ inhibitor SD208. To induce cell invasion, 600 μL 10% FBS-containing DMEM was loaded into the lower chamber. After overnight incubation, the remaining non-invading cells in the upper chamber were removed with a cotton swab. The invading cells adhering to the lower surface were fixed and stained using crystal violet. The stained cells were counted in ten randomly selected fields, under an inverted light microscope (IX71), at ×4 magnification.

### Cell proliferation assay

Cells were seeded in 96-well plates at a density of 1 × 10^4^ cells/well, and were treated with various chemotherapeutic agents for either 48 or 72 h. Cell viability was measured with either AlamarBlue^®^ solution (Invitrogen) or WST-1 reagent (Beyotime, Shanghai, China) following the manufacturer’s instructions. Chemotherapeutic agents and chemicals dimethylsulfoxide (DMSO), bicalutamide, abiraterone, docetaxel, etoposide and hydrogen peroxide (H_2_O_2_) were purchased from Sigma. Charcoal dextran-stripped serum (CDSS) was obtained from Gemini (West Sacramento, CA, USA) and Enzalutamide was obtained from Selleck Chemicals (Houston, TX, USA).

### Flow-fluorescence in situ hybridisation (flow-FISH)

Flow-FISH was conducted to measure the telomere length of the cells. Flow cytometer calibration, cell fixation, staining protocol and normalisation were conducted using mouse lymphoma cells with known telomere lengths. In all, 5 × 10^5^ ALDH^+^ CD44^+^ CXCR4^+^ CD24^+^ cells, ALDH^−^ CD44^−^ CXCR4^−^ CD24^−^ cells or mouse lymphoma cells were washed in hybridisation buffer and resuspended in hybridisation solution containing formamide and 0.3 μg/ml FITC-conjugated pentose nucleic acid (PNA) probe. Control samples were incubated in hybridisation solution without a PNA probe. Lymphoma cells were distinguished from cell derivatives by immunostaining with CD45 antibody (Millipore). The DNA content was quantified using propidium iodide staining. Cells were gated at G0/G1 based on DNA content, and the telomere fluorescence intensity was calculated, as previously described [30]. Detections were conducted on an FACSCanto flow cytometer (Becton Dickinson, Franklin Lakes, NJ, USA).

### Statistical analysis

Statistical analyses were performed with SPSS 23.0 (SPSS Inc., Chicago, IL, USA). Unless stated otherwise, normally distributed data are presented as the mean ± standard deviation of at least three independent experiments. Multiple groups were compared by ANOVA, followed by post hoc analysis (S-N-K test). When categorical data were compared, a chi-square test was used. A two-tailed *P* value below 0.05 was considered statistically significant.

## Results

### HOXB9 is induced in orthotopic PCa tumours and is essential to PCa lung metastasis

**Fig. 1:**
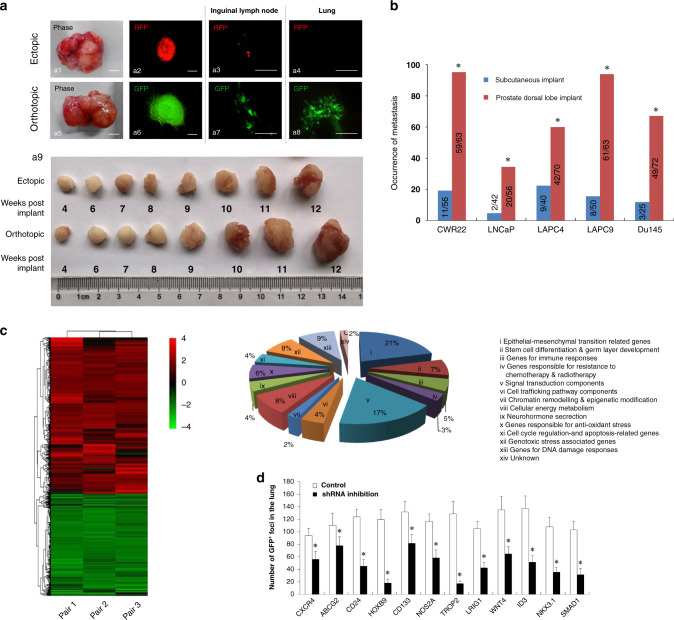
HOXB9 is one of the differentially expressed genes (DEGs) in mouse orthotopic prostate tumours, compared with ectopically implanted tumours. Six- to eight-week-old NOD/SCID mice were used. Prostate cancer (PCa) cells (CWR22, LNCaP, LAPC4, LAPC9 or Du-145) expressing green and red fluorescent proteins (GFP and RFP) were implanted into the dorsal lobe of the prostate and a subcutaneous area of the same mouse, respectively. Mice were sacrificed at 2 months after the initial appearance of tumours, and the primary tumours were immediately harvested. GFP or RFP fluorescence in primary tumours, as well as in metastatic foci in nine organs (bilateral lungs and kidneys, pancreas, liver, spleen, brain and bone marrow), were visualised using Nikon SMZ1500 whole-mount epifluorescence dissecting microscopy. Representative images of GFP- (a1–a4) and RFP-labelled (a5–a8) primary tumours (a1–a2, a5–a6), lymph node (a3, a7) and lung (a4, a8) metastases, as well as cell implant-derived xenografts on the timeline of tumour growth (a9) are shown in (**a**). Scale bars, 2 mm for a1 and a5; 100 µm for a2 and a6; 200 µm for a3, a4, a7 and a8. **b** The metastasis rate was calculated as the total number of metastatic foci detected in each group/(the number of detected organs × mouse number in each group). *P < 0.05 vs. the subcutaneous implant group. **c** CWR22-GFP cells with the highest metastasis rates were inoculated into the dorsal prostate and a subcutaneous area of the same mouse (*n* = 3). Microarray analysis was performed to identify the DEGs (*P* < 0.05, |fold change | ≥2) in the orthotopic tumours compared with the subcutaneous ones (Pair I and II), as well as in the GFP^+^-tumour cells isolated from the orthotopic tumours compared with those from ectopic tumours (Pair III) at 12 weeks post transplantation. All samples were run in triplicate. A representative heatmap of orthotopic tumours and GFP^+^-tumour cells derived from orthotopic tumours was obtained by hierarchical clustering. Red indicates the upregulated transcripts and green indicates the downregulated transcripts. The pie chart illustrates the classification of upregulated genes in orthotopic versus ectopic tumours. **d** Twelve CSC growth-related DEGs that were in the top 50 most upregulated DEGs were knocked down in CWR22-GFP cells using short hairpin RNAs, and cells with gene deficiencies were implanted into the mouse dorsal prostates (*n* = 12/group). At 12 weeks post-transplant, each mouse’s GFP^+^-pulmonary metastases were counted. Data are presented as the mean ± SD of the mean. *P < 0.05 vs. control.

To compare the metastatic potentials of the orthotopically and ectopically implanted PCa tumours, we inoculated GFP-labelled PCa cells (CWR22, Du-145, LNCaP, LAPC4 and LAPC9) and RFP-labelled cells into the dorsal lobe of the prostate and a subcutaneous area of the same mouse, respectively. Metastases in nine organs, including bilateral lungs and kidneys, pancreas, liver, spleen, brain and bone marrow, were identified by detecting GFP or RFP (Fig. 1a and Supplementary Table 3). For all five kinds of PCa cells, the metastasis rates for dorsal prostate implantation were significantly higher than for those with subcutaneous implantation (Fig. 1b and Supplementary Table 3). To reveal the characteristic gene expression pattern underlying the increased metastatic potential of orthotopic PCa tumours, we next implanted CWR22-GFP cells with the highest metastasis rate into the dorsal prostate and a subcutaneous area of the same mouse. Then, we performed microarray analysis to identify the DEGs (*P* < 0.05, fold change ≥2) between orthotopic and subcutaneous tumours. The 791 identified DEGs (602 upregulated and 189 downregulated) were functionally classified into 11 categories (I–XI; Fig. 1c), among which three categories (I, II and III) with the greatest fold changes were associated with tumour metastasis, CSC growth and inflammation/immunity, respectively. The top 50 upregulated genes are provided in an excel list (Supplementary Table 4). In addition, among the 50 most upregulated DEGs, 12 were associated with CSC growth regulation: CXCR4, CD133, ABCG2, CD24, HOXB9, NOS2A, TROP2, LRIG1, WNT4, ID3, NKX3.1 and SMAD1. To confirm whether these DEGs had contributed to the metastasis originating from the orthotopic tumours, we knocked down individual DEGs in CWR22-GFP cells which were then inoculated into mouse dorsal prostates and the lung metastases were detected. As shown in Fig. 1d, the numbers of pulmonary metastases significantly decreased in the mice which had received CSC-related gene-deficient CWR22 transplant cells, compared with the control. This suggests that these CSC-related DEGs are essential to CWR22 metastasis. Among them, knockdown of TROP2 and HOXB9 had the greatest inhibitory effects (87% and 85%, respectively) on CWR22 lung metastasis, suggesting that TROP2 and HOXB9 regulate PCa metastasis.

### HOXB9 is overexpressed in PCa and positively correlated with Gleason scores

**Fig. 2:**
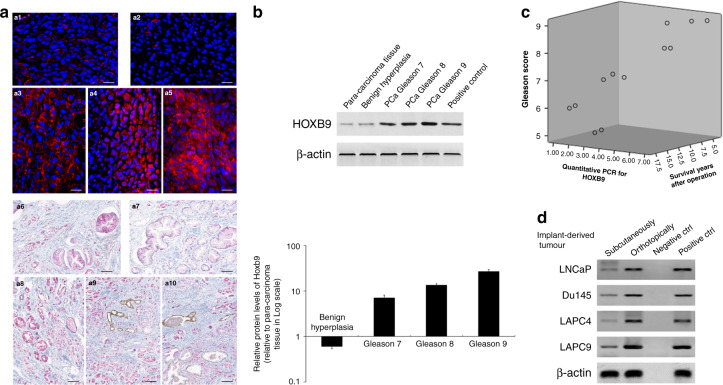
HOXB9 expression in tumour tissues derived from PCa patients. The endogenous expression of HOXB9 protein was detected in para-carcinoma and PCa tissues with Gleason scores 7–9 from 32 patients with primary PCa, as well as prostate tissues from patients with benign prostate hyperplasia, using immunofluorescent staining (**a**, a1–a5), immunohistochemical staining (**a**, a6–a10) and western blot assay (**b**), respectively. **a** Morphological staining for HOXB9. a1–a5, Immunofluorescence study on HOXB9 (red) with nucleus counterstained by DAPI (blue); a6–a10, Immunohistochemical staining for HOXB9 (pink colour in the cytoplasm), as well as P63 (brown colour in the nucleus) and cytokeratin 18 (brown colour in the cytoplasm), with nucleus counterstained by hematoxylin (blue). a1–a10, representative images derived from para-carcinoma tissue (a1, a6), benign hyperplasia (a2, a7), prostate cancer tissue at Gleason score 7 (a3, a8), 8 (a4, a9) and 9 (a5, a10), respectively. Scale bars, 20 µm for a1–a5, 100 µm for a6–a10. **b** Western blotting for HOXB9 in tissues as indicated (upper panel) and quantitative analysis (lower panel, *n* = 8 for each group). The positive control refers to breast cancer tissue derived from a breast cancer patient at the clinical stage of T4N2M0. **c** mRNA expression of HOXB9 was found to positively correlate with the Gleason score and negatively correlate with survival years after operation. Correlation analysis between HOXB9 expression levels and developmental stages of prostate cancer as well as prognosis suggested that HOXB9 mRNA levels were positively correlated with prostate cancer Gleason scores (*n* = 12, *r* = 0.918; *P* < 0.01), and negatively correlated with prognostic survival years after radical prostatectomy (*n* = 12, *r* = -0.917; *P* < 0.01). **d** Western blotting analysis on HOXB9, indicating the expression of HOXB9 is upregulated in the orthotopic vs subcutaneous tumours originated by LNCaP, DU154, LAPC4 and LAPC9 cells at 12 weeks post injection. Negative ctrl, negative controls (in which lane-specific antibodies were replaced with saline); positive ctrl, positive controls derived from human prostate cancer tissue of Gleason 8 after prostatectomy.

Since HOXB9 has been implicated in lung metastasis of breast carcinoma [25], we investigated the role of HOXB9 in PCa tissues. The results showed that HOXB9 was abundant in PCa tissues, but weak or not expressed in para-carcinoma and benign prostate hyperplasic tissues (Fig. 2a). These findings were consistent with HOXB9 protein expression in PCa tissues (Fig. 2b). Of note, the upregulation of HOXB9 mRNA was correlated with increased Gleason scores (Fig. 2c). As shown in Fig. 2d, the expression of HOXB9 was also upregulated in the orthotopic and subcutaneous tumours originated by LnCaP, DU154, LAPC4 and LPC9 cells. These findings suggested that HOXB9 may be involved in PCa development.

### HOXB9 regulates various tumour metastasis- and CSC growth-related genes through TGFβ signalling

**Fig. 3:**
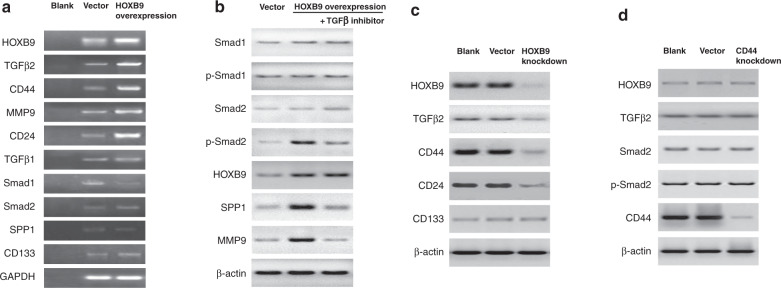
HOXB9 regulates various tumour metastasis- and cancer stem cell (CSC) growth-related genes via transforming growth factor beta (TGFβ) signalling. Du-145-GFP cells were stably transfected with myc-coupled vectors expressing HOXB9. **a** Semi-quantitative PCR was carried out to determine the mRNA expression of HOXB9, TGFβ2, CD44, MMP9, CD24, TGFβ1, Smad1, Smad2, osteopontin (SPP1) and CD133. GAPDH was used as an internal control. **b** Western blot analysis was performed to determine the protein levels of total Smad1, phospho (p)-Smad1, Smad2, p-Smad2, HOXB9, SPP1 and MMP9 in HOXB9-overexpressed Du-145-GFP cells, in the absence or presence of TGFβ inhibitor SD208 (5 μM) for 2 h. β-actin was used as an internal control. **c** HOXB9, TGFβ2, CD44, CD24 and CD133 protein levels were determined in Du-145-GFP cells with HOXB9 knockdown. **d** HOXB9, TGFβ2, total Smad2, p-Smad2 and CD44 protein levels were determined in Du-145-GFP cells with CD44 knockdown. An empty vector was used as a negative control.

To investigate the mechanisms underlying the role of HOXB9 in PCa metastasis, we examined the effects of HOXB9 on the expression of a panel of representative genes from the top two DEG categories, namely tumour metastasis- and CSC growth-related genes, in orthotopic implantation tumours. The results showed that the mRNA expression levels for TGFβ2, CD44, MMP9 and CD24 were significantly elevated in HOXB9-overexpressed PCa cells, compared with those in empty vector-transfected cells. Meanwhile, no significant difference in the mRNA expression of TGFβ1, Smad1, Smad2, osteopontin (SPP1) or CD133 was observed (Fig. 3a). However, the p-Smad2 protein levels (but not the total Smad2), SPP1 and MMP9 increased significantly in response to HOXB9 overexpression (Fig. 3b). Treatment with TGFβ inhibitor reversed these effects without affecting the HOXB9 protein expression (Fig. 3b). These data suggest that HOXB9 promotes TGFβ2/Smad2, thus upregulating protein expression of downstream target genes such as SPP1 and MMP9. In addition, the knockdown of HOXB9 inhibited the protein expressions of TGFβ2, CD44 and CD24, compared with the control group (Fig. 3c). Meanwhile, CD44 knockdown had no effect on TGFβ2, Smad2 or p-Smad2 protein expression (Fig. 3d). This suggests that HOXB9 mediates the TGFβ2/Smad2/CD44 signalling cascade.

### ALDH^+^CD44^+^CXCR4^+^CD24^+^- PCa cells exhibit stemness features in vitro and in vivo

**Fig. 4:**
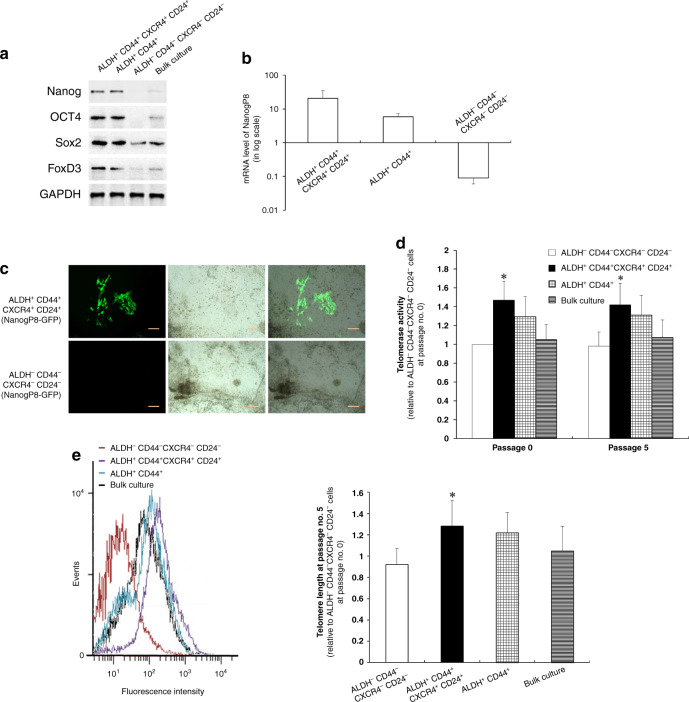
Pluripotency-related features of ALDH^+^CD44^+^CXCR4^+^CD24^+^-CWR22 cells. **a** As shown by Western blotting analysis, ALDH^+^ CD44^+^ CXCR4^+^ CD24^+^-CWR22 cells exhibit more robust expression of major pluripotency regulators such as Nanog, OCT4, Sox2 and FoxD3, as compared to ALDH^+^ CD44^+^-CWR22 cells, ALDH^−^ CD44^−^ CXCR4^−^ CD24^−^-CWR22 cells and bulk culture. **b** Quantitative RT-PCR analysis demonstrated that ALDH^+^ CD44^+^ CXCR4^+^ CD24^+^-PCa cells have much higher expression level of *NanogP8* mRNA, which was thought to be a pluripotency marker gene in cancer stem cells, as compared to controls run in parallel. **c** Cells labelled with reporter gene NanogP8-GFP, 1 × 10^3^ ALDH^+^CD44^+^CXCR4^+^CD24^+^ -CWR22 cells could be differentiated into a mixed bulk culture in a “salt and pepper” pattern in vitro; whereas ALDH^-^CD44^-^CXCR4^-^CD24^-^-PCa cells exhibited no NanogP8 expression under routine culture conditions. Scale bar, 10 µm. **d** The telomerase activity was measured by quantitative qRT-PCR. **e** Telomere fluorescence intensities and telomere length were measured by flow-fluorescence in situ hybridisation. Right panel, quantification of the left panel.

By comparing the gene expression profiles of orthotopic and subcutaneous PCa tumours, we selected five widely used CSC markers (ALDH, CD44, CXCR4, α2β1 and CD24) to sort candidate PCSCs from orthotopic PCa tumours. The five types of cell compartments of PCSCs were CD44^+^-, CD44^+^α2β1^+^-, ALDH^+^CD44^+^, ALDH^+^CD44^+^α2β1^+^- and ALDH^+^CD44^+^CXCR4^+^CD24^+^- PCa cells. The results of the tumorsphere and colony-formation assays showed more floating tumorspheres and colonies formed by ALDH^+^CD44^+^CXCR4^+^CD24^+^- PCa cells cultured in CDSS-supplemented medium to mimic androgen deprivation than those in the control groups (Supplementary Fig. 1A–D). This indicated the enhanced tumour-initiation abilities of ALDH^+^CD44^+^CXCR4^+^CD24^+^- PCa cells in vitro, under androgen-deprived conditions in vitro. In addition, in the tumour tissues derived from ALDH^+^CD44^+^CXCR4^+^CD24^+^-PCa cells implanted into the dorsal lobe of NOD/SCID mouse prostates for 6 weeks, we observed the presence of CXCR4^–^ and (or) CD24^–^ cells (Supplementary Fig. 1E), suggesting that ALDH^+^CD44^+^CXCR4^+^CD24^+^-PCa cells are pluripotent and can differentiate into multiple progenies in vivo. Furthermore, we found that as few as ten ALDH^+^CD44^+^CXCR4^+^CD24^+^-PCa cells, but not other subsets, could develop a palpable tumour within 100 days of implantation. Inoculation with 10^2^ or 10^3^ ALDH^+^CD44^+^CXCR4^+^CD24^+^-PCa cells produced the greatest tumour mass within the shortest time, as compared with three other sorted subpopulations (Supplementary Fig. 2A, B). In addition, the highest count of ALDH^+^CD44^+^CXCR4^+^CD24^+^ subset-derived metastatic foci was also observed in multiple organs (Supplementary Fig. 2C). These results indicate that ALDH^+^CD44^+^CXCR4^+^CD24^+^-PCa cells have tumorigenic and metastatic potential superior to other isogenic cells. Most protein expression levels of the major pluripotency regulators (Nanog, OCT4, Sox2 and FoxD3) [31–34], were found to increase significantly in ALDH^+^CD44^+^CXCR4^+^CD24^+^-PCa cells, compared with those in the ALDH^−^CD44^−^CXCR4^−^CD24^−^ subsets (Fig. 4a).

The overexpression of NanogP8, an important paralog of the Nanog family [35], was observed in ALDH^+^CD44^+^CXCR4^+^CD24^+^-PCa cells. This was evidenced by the dramatically upregulated mRNA (Fig. 4b) and protein expression (Fig. 4c), compared with the ALDH^−^CD44^−^CXCR4^−^CD24^−^ subpopulation. In addition, the ALDH^+^CD44^+^CXCR4^+^CD24^+^-PCa cells exhibited increased telomerase activity and longer telomeres than the ALDH^−^CD44^−^CXCR4^−^CD24^−^-PCa cells, and these trends persisted for at least five passages (Fig. 4d, e). The increased telomerase activity and longer telomeres are two important factors for maintaining stem cell pluripotency [36]. These findings suggest that specific CSC markers may be correlated with tumorigenesis and metastasis. Collectively, our data demonstrate that orthotopic PCa tumour-derived ALDH^+^CD44^+^CXCR4^+^CD24^+^ cell compartment is a maintainable subpopulation of pluripotent PCSCs with more tumorigenic and metastatic potential than other isogenic PCa cells.

### ALDH^+^ CD44^+^ CXCR4^+^ CD24^+^-PCa cells are resistant to surgical and chemical castration

**Fig. 5:**
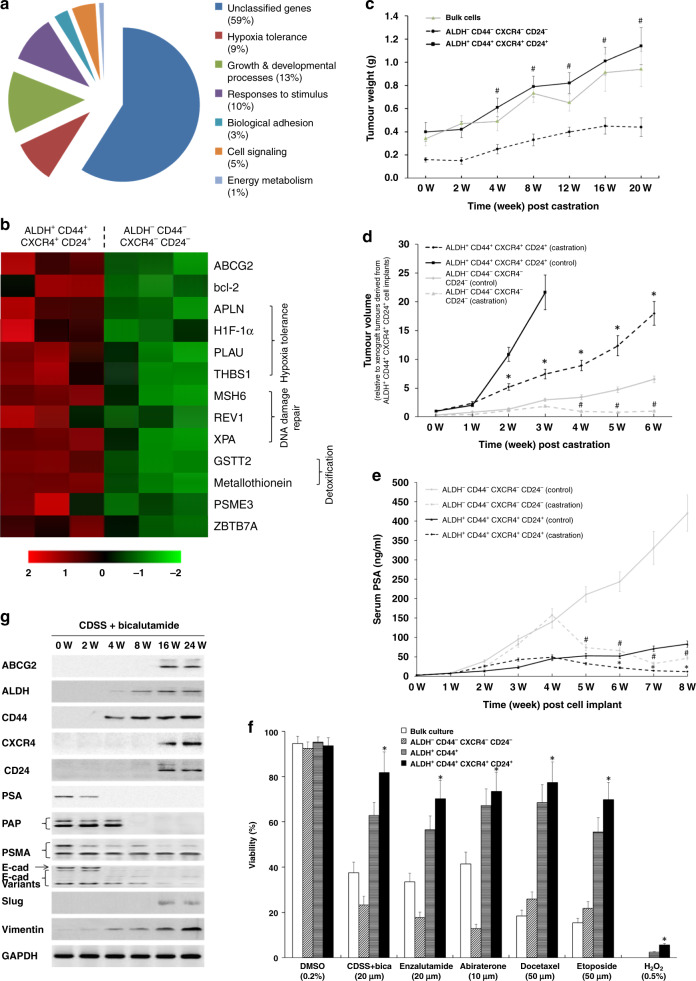
Castration resistance of ALDH^+^CD44^+^CXCR4^+^CD24^+^-PCa cells. **a** A microarray analysis was performed to identify the DEGs (*P* < 0.05, fold change ≥2) between ALDH^+^CD44^+^CXCR4^+^CD24^+^- and ALDH^−^CD44^−^CXCR4^−^CD24^−^-CWR22 cells. The pie chart illustrates how the DEGs were classified. **b** A heatmap obtained by hierarchical clustering shows the altered genes (>2.0-fold) between these two subsets of cells. Purified ALDH^+^CD44^+^CXCR4^+^CD24^+^- and ALDH^−^CD44^−^CXCR4^−^CD24^−^-CWR22 cells were subcutaneously implanted into male NOD-SCID mice. When tumours became palpable, the mice were treated with castration and bicalutamide, in combination. Tumour weights (**c**), tumour volumes (**d**) and serum PSA levels (**e**) were determined at different time points as indicated, respectively. *P < 0.05 vs. ALDH^+^ CD44^+^ CXCR4^+^ CD24^+^ (control); ^#^*P* < 0^.^05 vs. ALDH^−^ CD44^−^ CXCR4^−^ CD24^−^ (control). **f** CWR22 xenograft^-^derived bulk cells, ALDH^−^CD44^−^CXCR4^−^CD24^−^-, ALDH^+^CD44^+^-, and ALDH^+^CD44^+^CXCR4^+^CD24^+^-cells were plated in 96-well plates at a density of 1 × 10^4^/well and treated with different anti-androgens (as indicated) and chemotherapeutic agents, with 0.2% DMSO and 0.5% H_2_O_2_ were used as negative and positive controls, respectively. After 48 h of treatment, cells were incubated with alamarBlue solution for 4 h, and cell viability was measured with excitation wavelength at 530–560 nm and emission wavelength at 590 nm using a TECAN Infinite 200 PRO microplate reader. *P < 0.05 vs. ALDH^−^ CD44^−^ CXCR4^−^ CD24^−^ cells as well as bulk culture. **g** Western blot analysis was conducted to show time-dependent changes in phenotypic molecules and epithelial–mesenchymal transition-associated genes in ALDH^−^ CD44^−^ CXCR4^−^ CD24^−^ cells derived from CWR22 xenograft in the presence of CDSS and bicalutamide (10 µM).

To investigate the mechanism underlying the superior abilities of tumour initiation, formation, invasion and metastasis of the ALDH^+^ CD44^+^ CXCR4^+^ CD24^+^ subpopulation, we performed microarray analysis to identify the DEGs between the ALDH^+^ CD44^+^ CXCR4^+^ CD24^+^- and ALDH^−^ CD44^−^ CXCR4^−^ CD24^−^-PCa cells. The identified DEGs, such as ABCG2, bcl-2 and APLN, were functionally classified into seven categories (Fig. 5a, b). In the ectopic tumour model receiving castration and bicalutamide therapy, massive solid tumours were observed in ALDH^+^CD44^+^CXCR4^+^CD24^+^-cell-injected mice, whereas the ALDH^−^CD44^−^CXCR4^−^CD24^−^ cells yielded smaller tumours (Fig. 5c, d). It was noted that the serum PSA level in ALDH^+^ CD44^+^ CXCR4^+^ CD24^+^-cell-injected mice, either in the castration group or in control, was significantly lower than that in ALDH^−^ CD44^−^ CXCR4^−^ CD24^−^-cell-injected mice, respectively, indicating a similar scenario with clinical setting (Fig. 5e). These data suggest that ALDH^+^CD44^+^CXCR4^+^CD24^+^-cell-derived tumours may be less sensitive to androgen deprivation/antiandrogen therapy than those derived from ALDH^−^CD44^−^CXCR4^−^CD24^−^ cells. Indeed, the cell viabilities of ALDH^+^CD44^+^CXCR4^+^CD24^+^ cells were significantly higher than those of other subpopulations, in response to a broad spectrum of chemotherapeutic agents (Fig. 5f). These data suggest that ALDH^+^CD44^+^CXCR4^+^CD24^+^ cells are resistant to surgical/chemical castration therapy. To reveal the mechanism underlying cell castration resistance, we next isolated ALDH^−^CD44^−^CXCR4^−^CD24^−^ cells from CWR22 xenografts, which were then exposed to anti-androgens (CDSS plus bicalutamide) at different time points (0, 2, 4, 8, 16 and 24 weeks). We then determined the protein expression of a panel of PCSC markers, differentiation markers, and epithelial–mesenchymal transition (EMT)-related genes. As shown in Fig. 5g, the PCSC marker protein levels (ABCG2, ALDH, CD44, CXCR4 and CD24) were time-dependently elevated. This suggests the antiandrogen-induced phenotypic conversion of ALDH^−^CD44^−^CXCR4^−^CD24^−^ cells into ALDH^+^CD44^+^CXCR4^+^CD24^+^ cells. In addition, the time-dependent regulations of EMT-related genes (downregulation of E-cadherin, and upregulation of Slug and Vimentin) and downregulation of differentiation markers (PSA, PAP and PSMA) indicated a gradual EMT induction by anti-androgens. Collectively, these results suggest that chemoresistance in PCa may at least be partially attributable to the antiandrogen-induced phenotypic conversion and EMT in tumour cells.

### HOXB9 is more abundant in human high-grade PCa tissues which harbour ALDH^+^ CD44^+^ CXCR4^+^ CD24^+^ subpopulation and silencing HOXB9 enhanced the sensitivity to various chemotherapeutic agents and decreased the metastatic ability of ALDH^+^CD44^+^CXCR4^+^CD24^+^ cells

**Fig. 6:**
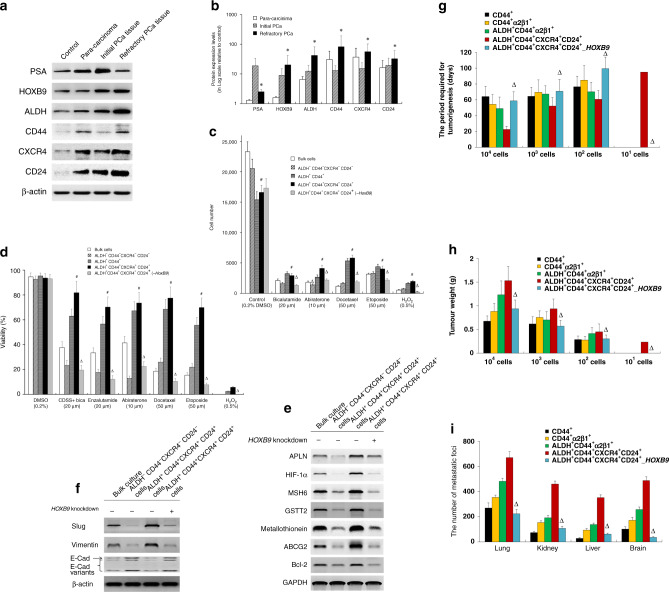
The ALDH^+^CD44^+^CXCR4^+^CD24^+^ subpopulation in human PCa tissues. **a** Western blot analysis was performed to determine the protein expressions of PSA, HOXB9, ALDH, CD44, CXCR4 and CD24 in the controls (PCa tissue with Gleason score 6), para-carcinoma (2 mm away from PCa tissue), initial PCa tissue (derived from PCa at first diagnosis via radical prostatectomy) and refractory PCa tissue (derived after recurrence), respectively. β-actin was used as an internal control. **b** Quantification of (**a**). *P < 0.05 vs. initial PCa tissues. (*n* = 6). **c** Human PCa tissue was subcutaneously implanted into NOD-SCID mice to establish a patient-derived xenograft (PDX) model. Subsets of cells (as indicated) were derived from the PDX model and seeded in 96-well plates (1 × 10^4^ cells/well) and treated with different anti-androgens (as indicated) and chemotherapeutic agents, with 0.2% DMSO and 0.5% H_2_O_2_ were used as negative and positive controls, respectively. After 48 h of treatment, cells were incubated with alamarBlue solution for 4 h, and cell viability was measured with excitation wavelength at 530–560 nm and emission wavelength at 590 nm using a TECAN Infinite 200 PRO microplate reader. **d** Subsets of cells (as indicated) were derived from the PDX model and seeded in 96-well plates (1 × 10^4^ cells/well). Cells were treated with different chemotherapeutic drugs, as indicated, for 72 h. Then, a WST-1 proliferation assay was performed. The absorbance was measured at 450 nm using a microplate reader. ^#^*P* < 0.05 vs. bulk cells, as well as ALDH^–^CD44^–^CXCR4^–^CD24^–^ -cells (*n* = 12)^; Δ^*P* < 0.05 vs. ALDH^+^CD44^+^CXCR4^+^CD24^+^-cells (*n* = 12). **e** ALDH^+^ CD44^+^ CXCR4^+^ CD24^+^-cells were isolated from PDX tumours at 8 weeks post implantation, before they were subject to *HOXB9* knockdown. Then cells at 1 × 10^6^ (together with 1 × 10^6^ HS-5 cells to facilitate tumour formation) were subcutaneously injected into the NOD/SCID mouse. Derived tumours at 12 weeks post injection were subject to Western blotting analysis of the expression of APLN, HIF-1α, MSH6, GSTT2, metallothionein, ABCG2 and Bcl-2). GAPDH was used as an internal control. **f** Western blotting analysis of the expression of epithelial–mesenchymal transition-associated genes (Slug, Vimentin and E-cadherin). β-actin was used as an internal control. (G-I) CD44^+^-, CD44^+^ α2β1^+^-, ALDH^+^ CD44^+^ α2^β^1^+^- and ALDH^+^ CD44^+^ CXCR4^+^ CD24^+-^PCa cells were obtained from orthotopic CWR22 tumours by FACS using the respective antibodies, whereas *HOXB9*-silenced ALDH^+^ CD44^+^ CXCR4^+^ CD24^+^-PCa cells were derived from ALDH^+^ CD44^+^ CXCR4^+^ CD24^+^-cells. Orthotopic tumour models were established using these subsets of cells, respectively. Mice were sacrificed at week 14 after inoculation. The time for developing a palpable tumour (**g**), tumour weights (**h**) and the number of metastatic foci (**i**) were recorded (*n* = 12). ^Δ^*P* < 0.05 vs. ALDH^+^ CD44^+^ CXCR4^+^ CD24^+^ cells-based implantation.

To explore this study’s clinical relevance, we determined the protein expression of ALDH, CD44, CXCR4 and CD24 in human PCa tissues. As shown in Fig. 6a and b, when compared with those in low-grade PCa tissues with Gleason scores of 6 (control), the ALDH, CD44, CXCR4 and CD24 protein levels were generally elevated in para-carcinoma tissues. They were also elevated in initial PCa tissues collected at first diagnosis or after recurrence via radical prostatectomy, as well as in refractory PCa tissues. Of these, the protein levels in the refractory PCa tissues were the highest, suggesting the presence of ALDH^+^CD44^+^CXCR4^+^CD24^+^ tumour cells in human PCa tissues. The same trend was also observed in HOXB9 protein expression. This was consistent with the in vitro observations, as well as those from mouse models. To confirm the existence of ALDH^+^CD44^+^CXCR4^+^CD24^+^ cells in human PCa tissues, we established a PDX model by subcutaneously implanting human PCa tissues into NOD-SCID mice. As expected, we isolated ALDH^+^CD44^+^CXCR4^+^CD24^+^ cells from the tumour derived from the PDX model by cell sorting. In addition, the results of a cell proliferation assay showed that there were more PDX model-derived ALDH^+^CD44^+^CXCR4^+^CD24^+^ cells than PDX model-derived bulk cells or ALDH^−^CD44^−^CXCR4^−^CD24^−^ cells when there was exposure to various chemotherapeutic drugs. Also, silencing HOXB9 in ALDH^+^CD44^+^CXCR4^+^CD24^+^ cells significantly improved their sensitivities to various chemotherapeutic agents (Fig. 6c, d). Tumours derived from HOXB9 knockdown ALDH^+^CD44^+^CXCR4^+^CD24^+^ cells exhibited significantly decreased expression of hypoxia tolerance-related genes (APLN, HIF-1α), DNA damage repair-related gene (MSH6), detoxification-related genes (GSTT2, metallothionein), chemoresistant gene ABCG2, and anti-apoptotic gene Bcl-2 (Fig. 6e), and significantly decreased levels of epithelial–mesenchymal transition-associated genes (Slug and Vimentin) (Fig. 6f), as compared to those derived from intact ALDH^+^ CD44^+^ CXCR4^+^ CD24^+^-cell injection. Orthotopic tumour models were developed from CD44^+^, CD44^+^ α2β1^+^, ALDH^+^ CD44^+^ α2β1^+^ and ALDH^+^ CD44^+^ CXCR4^+^ CD24^+^ cells with or without HOXB9 knockdown. The time for developing a palpable tumour in HOXB9 knockdown ALDH^+^CD44^+^CXCR4^+^CD24^+^ cells was significantly longer than that of ALDH^+^ CD44^+^ CXCR4^+^ CD24^+^ cells (Fig. 6g), and the tumour weights (Fig. 6h), and the number of metastatic foci (Fig. 6i) were significantly decreased in HOXB9 knockdown ALDH^+^CD44^+^CXCR4^+^CD24^+^ cells compared with those of ALDH^+^ CD44^+^ CXCR4^+^ CD24^+^ cells. Taken together, these finding indicated that HOXB9 is highly expressed in human high-grade PCa which is enriched with ALDH^+^ CD44^+^ CXCR4^+^ CD24^+^ cells and silencing HOXB9 significantly improved the sensitivity to various chemotherapeutic agents and decreased the metastatic ability of ALDH^+^CD44^+^CXCR4^+^CD24^+^ cells.

### HOXB9/TGFβ2 contributes to the superior tumorigenic and metastatic potential of the ALDH^+^ CD44^+^ CXCR4^+^ CD24^+^ subpopulation derived from the PCa xenograft

To explore whether the HOXB9/TGFβ2 signalling is involved in the superior tumorigenic and metastatic potential of the ALDH^+^CD44^+^CXCR4^+^CD24^+^ subset, we determined the protein expressions of HOXB9 and TGFβ2 in different cell subsets isolated from CWR22 cell xenograft. As shown in Supplementary Fig. 3A, the protein levels of both HOXB9 and TGFβ2 were elevated in ALDH^+^ CD44^+^ CXCR4^+^ CD24^+^ cells, compared with the unsorted and other subsets of cells. Furthermore, TGFβ inhibitor significantly retarded tumour formation, while reducing cell migration and invasion abilities, tumour mass, and the number of metastatic foci derived from orthotopically implanted ALDH^+^ CD44^+^ CXCR4^+^ CD24^+^ subpopulation (Supplementary Fig. 3B–F). This suggests that the high tumorigenic and metastatic abilities of ALDH^+^CD44^+^CXCR4^+^CD24^+^ cells are at least partially attributable to the HOXB9/TGFβ signalling.

## Discussion

This study compared the gene expression profiles of orthotopic and ectopic tumours formed by PCa cells in mice. We selected HOXB9 as a candidate factor responsible for the extensive metastases originating from orthotopic tumours. HOXB9 is in the HOX transcription factor family and was overexpressed in many types of tumours [37–40]. Previous studies have shown that HOXB9 overexpression may promote distal metastasis, and is correlated with clinical outcomes in breast, colon, and lung cancers [25, 40, 41], which links HOXB9’s biological function to solid tumour invasion and metastasis. However, the role of HOXB9 in PCa remains unclear. In our study, we confirmed that HOXB9 was abundantly expressed in PCa tissues and that the HOXB9 overexpression was also correlated with increased Gleason scores and poor overall patient survival. This suggests that HOXB9 may be a prognosis biomarker for PCa patients. Our data demonstrated that HOXB9 knockdown mitigated the number of lung metastatic foci in the orthotopic PCa tumour models. Mechanistically, HOXB9 could alter the expression of a panel of CSC growth- and metastasis-related genes, as well as regulate the metastatic behaviour of ALDH^+^CD44^+^CXCR4^+^CD24^+^ -PCa cells, via TGFβ signalling. Collectively, these findings suggest that HOXB9 is essential for PCa to metastasis.

EMT is a process in which adherent epithelial cells are converted into migratory mesenchymal cells capable of invading the extracellular matrix, which plays a critical role in cancer metastasis [42]. TGFβ signalling is a classical molecular pathway associated with EMT [43]. It has been found that HOXB9 induces both TGFβ1 and TGFβ2 upregulation in normal human mammary epithelial cells. This enhances cell motility and mesenchymal phenotype acquisition [25], suggesting that HOXB9 may induce EMT by activating the TGFβ signalling. The TGFβ subfamily members mediate intracellular signalling via the Smad family [44]. Our results showed that the enforced expression of HOXB9 in PCa cells significantly increased TGFβ2 mRNA and p-Smad2 protein levels, while HOXB9 knockdown decreased TGFβ2 expression. Suppressing TGFβ receptor signalling with the specific inhibitor SD208 suppressed Smad2 phosphorylation without affecting HOXB9 expression. This suggests that HOXB9 is an upstream activator of TGFβ/Smad2 signalling. In addition, the HOXB9-induced upregulated expression of SSP1 and MMP9, two well-known cancer metastasis promoters [45, 46], was suppressed by TGFβ inhibitor. This suggests that SSP1 and MMP9 are downstream target genes of the HOXB9/TGFβ/Smad2 pathway in the regulation of cancer metastasis. Thus, we speculate that HOXB9 may activate TGFβ/Smad2 signalling, which in turn alters a panel of downstream target genes involved in cancer cell invasion and metastasis.

A previous study found that PCa cells expressing CSC marker CD44 are more tumorigenic and metastatic than isogenic CD44^−^ cells [13]. However, little is known about the underlying regulatory mechanism behind this. In this study, HOXB9 overexpression induced CD44 transcription, whereas HOXB9 knockdown suppressed CD44 protein expression. However, no changes were observed in the expression of HOXB9, TGFβ2 or p-Smad2 in response to CD44 knockdown. These data suggest that HOXB9 is an upstream regulator of CD44 and may be responsible for the enhanced metastatic potential of CD44^+^-PCa cells. To investigate the promotive role of HOXB9 in PCa metastasis, we isolated homogenous PCa subpopulations carrying different combinations of CSC markers from orthotopic PCa tumours. In the tumour models established with each subpopulation, we found that the combination of CSC markers synergistically reinforced the tumorigenic and metastatic abilities of PCa cells. Thus, they became potential mPCSCs. The preferential expression of the “stemness genes” Nanog, OCT4, Sox2 and FoxD3 [31–34] in the ALDH^+^CD44^+^CXCR4^+^CD24^+^ subpopulation may have endowed these cells with certain stem cell properties. Intriguingly, HOXB9 and TGFβ2 expression was significantly upregulated in PCa cells carrying different combinations of CSC markers, compared with those in unsorted cells (ALDH^+^CD44^+^CXCR4^+^CD24^+^ > CD44^+^α2β1^+^ >CD44^+^ in terms of HOXB9 or TGFβ2 protein levels). This may explain the synergism generated by combining CSC markers in PCa cells. The suppression of the TGFβ signalling with the inhibitor significantly suppressed the tumorigenic and metastatic abilities of ALDH^+^CD44^+^CXCR4^+^CD24^+^-PCa cells in vivo. This confirmed the importance of HOXB9/TGFβ signalling in PCa initiation and metastasis. Our results also demonstrated that ALDH^+^CD44^+^CXCR4^+^CD24^+^ cells were castration-resistant and that ALDH^−^CD44^−^CXCR4^−^CD24^−^ cells derived from CWR22 xenograft exhibited phenotypic conversion and EMT in a time-dependent manner in the presence of anti-androgens. This is consistent with a recent study [47]. HOXB9 and CSC markers were simultaneously overexpressed in human refractory PCa tissues, compared with low-grade PCa, para-carcinoma, and initial PCa tissues implying the involvement of HOXB9 signalling’s involvement in ALDH^+^CD44^+^CXCR4^+^CD24^+^-PCa cell-rendered castration resistance.

In conclusion, our study demonstrated the promotive role of HOXB9 in PCa tumorigenesis and metastasis through altering the expression of CSC- and metastasis-related genes and enhancing the tumorigenic/metastatic potential of mPCSCs via TGFβ signalling. HOXB9 overexpression was observed in patients’ primary PCa tissues. It was correlated with high-grade tumours and poor overall survival and involved in ALDH^+^CD44^+^CXCR4^+^CD24^+^-cell-rendered castration resistance. Therefore, HOXB9 may serve as a prognostic biomarker and a potential therapeutic target for refractory PCa treatment.

## Supplementary information

Supplementary figure and table legends

Supplementary Figure 1

Supplementary Figure 2

Supplementary Figure 3

Supplementary table 1

Supplementary table 2

Supplementary table 3

Supplementary table 4

Email response about co-first author

## Data Availability

All the original data which were available upon request were kept in the database management system within the Center for Medical Research and Innovation, Shanghai Pudong Hospital, Fudan University Pudong Medical Center.
