# Supplementary material for: Insights into homeobox B9: a propeller for metastasis in dormant prostate cancer progenitor cells
Source: Br J Cancer. 2021 Jul 10;125(7):1003–15. doi: 10.1038/s41416-021-01482-y (PMC8476533; doi:10.1038/s41416-021-01482-y)
Supplement: Supplementary file 1 — Supplementary figure and table legends [file 41416_2021_1482_MOESM1_ESM.docx]

**Supplementary Fig. 1**

**Supplementary Figure 1. Tumor initiation property and pluripotency of ALDH^+^ CD44^+^ CXCR4^+^ CD24^+^-PCa cells.** ALDH^+^ CD44^+^-, ALDH^+^ CD44^+^ CXCR4^+^ CD24^+^-, and ALDH^−^ CD44^−^ CXCR4^−^ CD24^−^-PCa cells were obtained from orthotopic CWR22- or LAPC4-implanted tumors by FACS using respective antibodies. (A, B) Tumor sphere formation assay. Each subset of PCa cells was seeded in an ultra-low attachment T25 flask at a density of 5×10^4^ cells/flask, and grown in RPMI supplemented with 10% charcoal dextran-stripped serum (CDSS). Tumor sphere formation was observed after 8 days of culture. In each flask, tumor spheres were counted at 10× magnification. Representative images are shown. Scale bar, 500 µm. Data are expressed as mean ± standard deviation (SD). * *p* < 0.05 vs. ALDH^+^ CD44^+^ CXCR4^+^ CD24^+^. (C, D) Colony formation assay. Each subset of LAPC4 cells was seeded in poly-L-ornithine- and laminin-coated 6-well plates at a density of 1×10^4^ cells/well, and maintained in IMDM supplemented with 15% CDSS. Colony formation was observed after 2 weeks of culture with medium refreshment every other day. The colonies formed were counted in each well at 10× magnification. Representative images are shown. Scale bar, 500 µm. Data are expressed as mean ± SD. * *p* < 0.05 vs. ALDH^+^ CD44^+^ CXCR4^+^ CD24^+^. (E) Pluripotency assay. 1×10^3^ ALDH^+^ CD44^+^ CXCR4^+^ CD24^+^-CWR22 cells were resuspended in a mixture of 20 μl medium and 20 μl Matrigel. The cell suspension was inoculated into the dorsal prostates of 6-8 weeks old NOD/SCID male mice supplemented with dihydrotestosterone pellets (0.2 mg/mouse embedded subcutaneously). Tumors were harvested after 6 weeks of inoculation, and FACS analysis was performed to isolate subpopulations of CWR22 cells. After *in vivo* implantation, the original ALDH^+^CD44^+^CXCR4^+^CD24^+^-PCa cells could be differentiated into subpopulations of P1, P2 and P3, as indicated.

**Supplementary Fig. 2**

**Supplementary Figure 2. Tumorigenic capacity of cell subpopulations derived from orthotopic implant tumors.** CD44^+^-, CD44^+^ α2β1^+^-, ALDH^+^ CD44^+^ α2β1^+^-, and ALDH^+^ CD44^+^ CXCR4^+^ CD24^+^-PCa cells were obtained from orthotopic CWR22 tumors by FACS using the respective antibodies, whereas *HOXB9*-silenced ALDH^+^ CD44^+^ CXCR4^+^ CD24^+^-PCa cells were derived from ALDH^+^ CD44^+^ CXCR4^+^ CD24^+^-cells. Orthotopic tumor models were established using these subsets of cells, respectively. Mice were sacrificed at week 14 after inoculation. The time for developing a palpable tumor (A), tumor weights (B), and the quantity of metastatic foci (derived from 1×10^3^ cell transplant; C) were recorded. * *p* < 0.05 vs. CD44^+^ cells-based implantation.

**Supplementary Fig. 3**

**Supplementary Figure 3.** **HOXB9/TGFβ2 signaling in the ALDH^+^CD44^+^CXCR4^+^CD24^+^ subpopulation derived from PCa xenografts.** (A) The protein expression of HOXB9 and TGFβ2 in different subsets of cells (as indicated) derived from orthotopic CWR22 implant tumors was determined by Western blot assay. β-actin was used as an internal control. The cell migratory (B) and invasive abilities (C) were evaluated in the presence or absence of TGFβ inhibitor SD208 (5 μM) using wound healing and transwell assays, respectively. Representative images of invading cells are shown in (C). Scale bar, 30 µm. 1×10^3^ ALDH^+^ CD44^+^ CXCR4^+^ CD24^+^ cells pretreated with TGFβ inhibitor SD208 (5 μM) or control were implanted into the dorsal prostates of mice, which were sacrificed at week 10 after implantation. The time for developing palpable tumors (D), tumor weights (E), and the quantity of metastatic foci in the lungs (F) were recorded. n=12. **P*<0.05 vs. the control.

**Supplementary table 1. shRNA sequences of 12 cancer stem cell (CSC) growth-related genes.**

**Supplementary table 2. Primer sequences for RT-PCR.**

**Supplementary table 3. Quantification of metastatic sites of CWR22, Du-145, LNCaP, LAPC4 and LAPC9 in the orthotopically and ectopically implanted mouse models.**

**Supplementary table 4. Top 50 up-regulated genes.**
