# Supplementary figures and images for: Insights into homeobox B9: a propeller for metastasis in dormant prostate cancer progenitor cells
Source: Br J Cancer. 2021 Jul 10;125(7):1003–15. doi: 10.1038/s41416-021-01482-y (PMC8476533; doi:10.1038/s41416-021-01482-y)

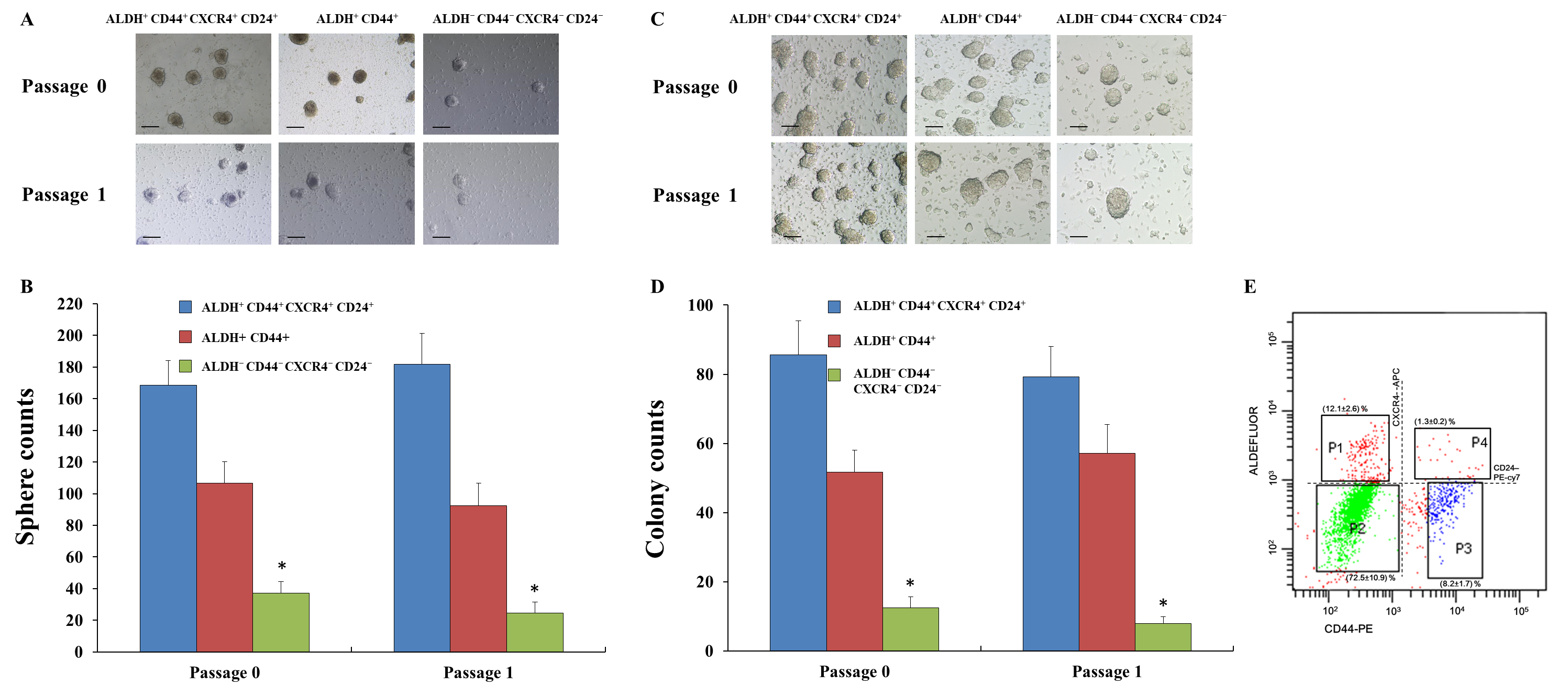

Supplement: Supplementary file 2 — Supplementary Figure 1 [file 41416_2021_1482_MOESM2_ESM.tif]

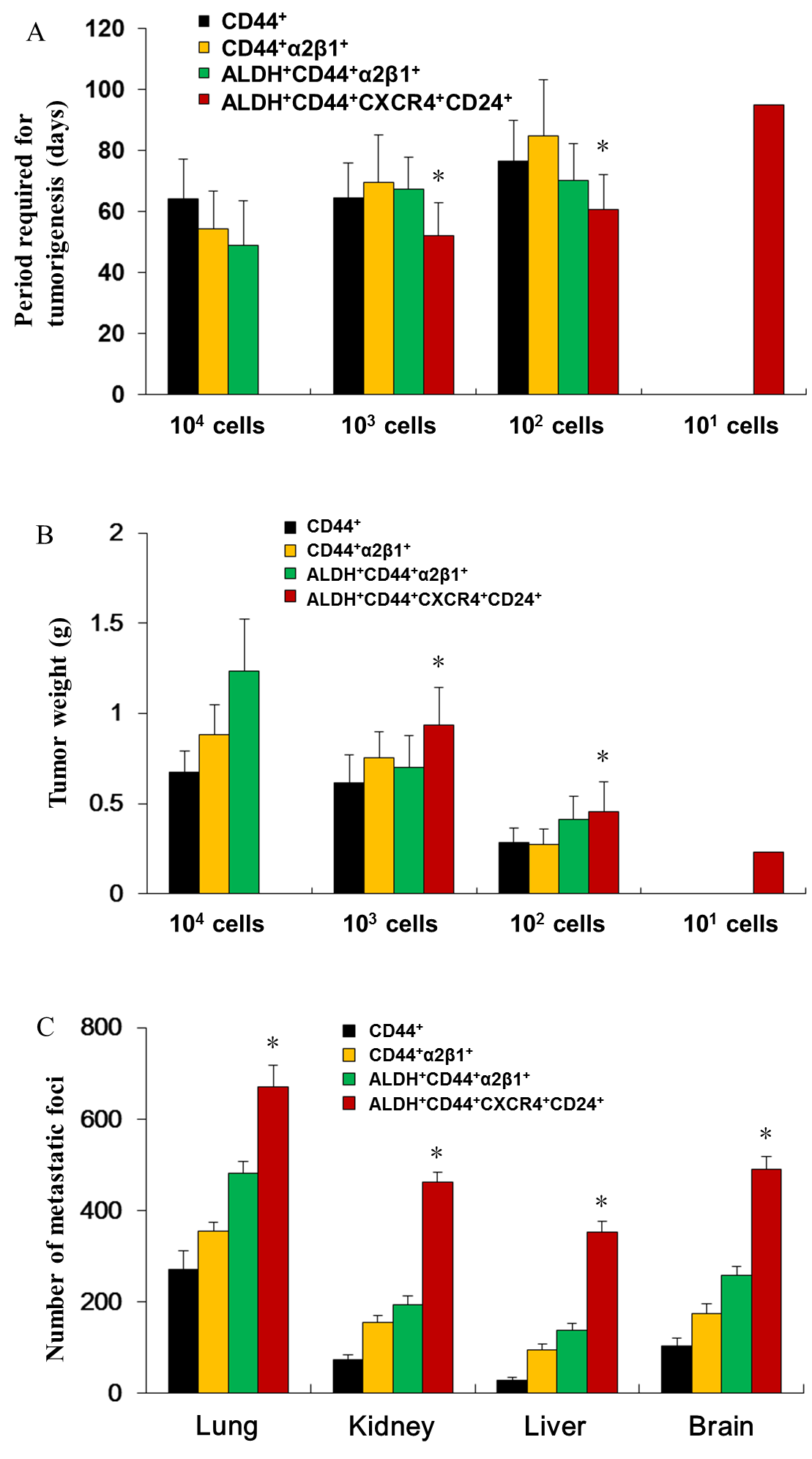

Supplement: Supplementary file 3 — Supplementary Figure 2 [file 41416_2021_1482_MOESM3_ESM.tif]

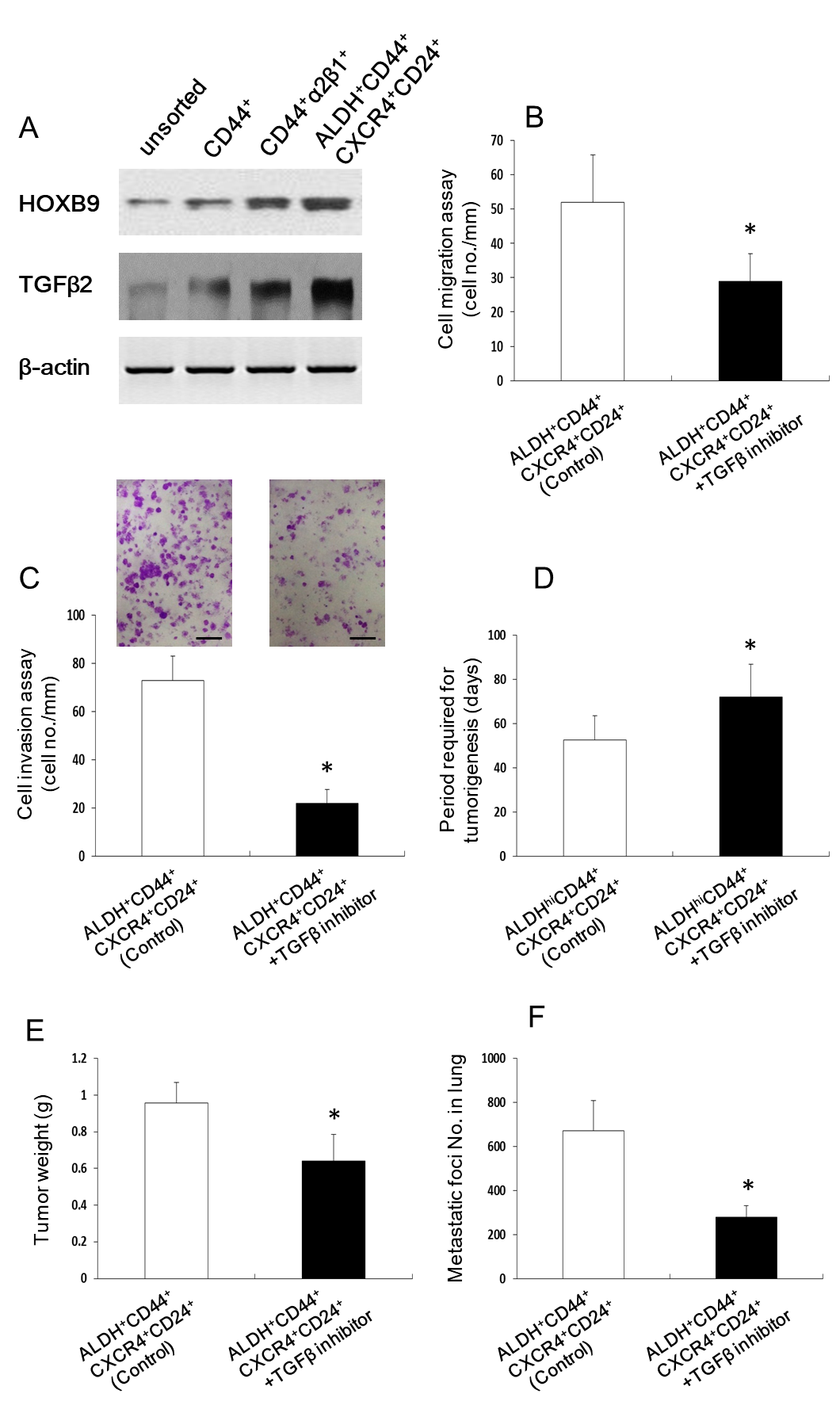

Supplement: Supplementary file 4 — Supplementary Figure 3 [file 41416_2021_1482_MOESM4_ESM.tif]
