# Supplementary material for: Insights into homeobox B9: a propeller for metastasis in dormant prostate cancer progenitor cells
Source: Br J Cancer. 2021 Jul 10;125(7):1003–15. doi: 10.1038/s41416-021-01482-y (PMC8476533; doi:10.1038/s41416-021-01482-y)
Supplement: Supplementary file 5 — Supplementary table 1 [file 41416_2021_1482_MOESM5_ESM.docx]

Supplementary Table 1 shRNA sequences

| Target | Accession | Transcribed RNAi sequence (5’–3’) | Scrambled RNAi sequence (5’–3’) |
| --- | --- | --- | --- |
| CXCR4 | NM_003467 | AGACUGAUGAAGGCCAGGAUU | AUUAUAGCAUUGGCAAUCUGC |
| CD133 | NM_001145847 | GUCCUUCCUAUAGAACAAU | UUCCUAACUAAGUCCUCAA |
| ABCG2 | NM_004827 | CCUGAGUUACGUUGUCCUU | AGUGAGUUACCGAGUCGUA |
| CD24 | NM_013230 | AACGUAACUGGUGCUGAUU | UAACGCGUCGUGCUGAACU |
| HOXB9 | NM_024017 | GGAGUUAGACUCACUCUCU | CACUCUAGAUCACUGUACU |
| NOS2A | NM_012611 | UUACACCUCUUUCGGGGGAC | UGGGACUACACCCUCUUUGA |
| TROP2 | NM_002353 | ACCUGUUGCUACCGGAGAU | UGCUCCGAGGAGAUGCUAC |
| LRIG1 | NM_015541 | UUCCGGAUGGAAAGGAAUCUU | UUUGAUGGCAACAAUAUCCAC |
| WNT4 | NM_030761 | CGUCUGUUUGGUUCUUACG | CCUGUAGCUUAAGAUCGUA |
| ID3 | NM_002167 | UUGAACAGUAGAGGUUGCUGU | UUAGCUAUAUGGUCGAACAUG |
| NKX3.1 | NM_006167 | AGGUCUUGUCUGCGAUAUU | AGCAUAUUUGUAGCAUGCC |
| SMAD1 | NM_001354817 | GUCCCGUUGGCUCAUUGAC | GUCGAUGGCUCACGAACUG |
| CD44 | NM_000610.3 | GACCTCTGCAAGGCTTTCAAT | ATTGAAAGCCTTGCAGAGGTC |

CXCR4, C-X-C chemokine receptor type 4; CD133, Prominin-1; ABCG2, ATP-binding cassette sub-family G member 2; CD24, signal transducer CD24; HOXB9, homeobox protein Hox-B9; NOS2A, inducible nitric oxide synthase; TROP2, tumor-associated calcium signal transducer 2; LRIG1, leucine-rich repeats and immunoglobulin-like domains protein 1; WNT4, wingless-related integration protein 4; ID3, DNA-binding protein inhibitor ID-3; NKX3.1, NK-3 transcription factor, locus 1; SMAD1, mothers against decapentaplegic homolog 1; CD44, CD44 molecule.
