# Supplementary material for: Insights into homeobox B9: a propeller for metastasis in dormant prostate cancer progenitor cells
Source: Br J Cancer. 2021 Jul 10;125(7):1003–15. doi: 10.1038/s41416-021-01482-y (PMC8476533; doi:10.1038/s41416-021-01482-y)
Supplement: Supplementary file 6 — Supplementary table 2 [file 41416_2021_1482_MOESM6_ESM.docx]

Supplementary Table 2 Primer sequences for RT-PCR

| Target genes | Forward (5´ – 3´) | Reverse (5´ – 3´) | Product (bp) |
| --- | --- | --- | --- |
| HOXB9 | CCGGCTACGGGGACAATAA | GGTGTAGGGACAGCGCTTTTT | 122 |
| TGFβ2 | CTGATCCTGCATCTGGTCACG | TGGGGGACTGGTGAGCTTC | 135 |
| CD44 | ACCCCAACTCCATCTGTGC | TTCTGGACATAGCGGGTG | 199 |
| MMP9 | CTGGACAGCCAGACACTAAAG | CTCGCGGCAAGTCTTCAGAG | 145 |
| CD24 | TCACTTTCCTCCTGAGGCTT | TCAAGAGTTGCTCAGGATGC | 125 |
| TGFβ1 | CAGCAACAATTCCTGGCGATA | AAGGCGAAAGCCCTCAATTT | 136 |
| Smad1 | AAACAGGGCGATGAAG | AGTGAGGAAACGGGTG | 390 |
| Smad2 | TTAAAGCACCTTGTGGAATC | AATAACGGAGAAGTGGGAATA | 330 |
| SPP1 | ATGATGGCCGAGGTGATAGT | ACCATTCAACTCCTCGCTTT | 134 |
| CD133 | GAAGAGCTTGCACCAACAAA | AGATGACCGCAGGCTAGTTT | 173 |
| GAPDH | ATCATCCCTGCCTCTACTGG | TTTCTAGACGGCAGGTCAGGT | 136 |
