# Supplementary material for: Insights into homeobox B9: a propeller for metastasis in dormant prostate cancer progenitor cells
Source: Br J Cancer. 2021 Jul 10;125(7):1003–15. doi: 10.1038/s41416-021-01482-y (PMC8476533; doi:10.1038/s41416-021-01482-y)
Supplement: Supplementary file 7 — Supplementary table 3 [file 41416_2021_1482_MOESM7_ESM.docx]

Supplementary Table 3 Quantification of metastatic sites of CWR22, Du-145, LNCaP, LAPC4 and LAPC9 in the orthotopically and ectopically implanted mouse models

|  | Implant mode | Model number | Lung  (L) | Lung  (R) | Kidney  (L) | Kidney  (R) | Pancreas | Liver | Spleen | brain | Bone marrow (femur) | Summation |
| --- | --- | --- | --- | --- | --- | --- | --- | --- | --- | --- | --- | --- |
| CWR22 | **Orthotopically** | 7 | 6 | 5 | 4 | 5 | 3 | 10 | 5 | 4 | 17 | 59 |
|  | **Ectopically** | 8 | 2 | undetected | 1 | undetected | 1 | 3 | 0 | 1 | 3 | 11 |
| Du-145 | **Orthotopically** | 8 | 4 | 3 | 3 | 3 | 3 | 9 | 6 | 8 | 10 | 49 |
|  | **Ectopically** | 5 | 1 | 0 | 1 | 0 | undetected | 1 | undetected | undetected | undetected | 3 |
| LNCaP | **Orthotopically** | 8 | 5 | undetected | 4 | undetected | 2 | 3 | 2 | 1 | 3 | 20 |
|  | **Ectopically** | 6 | 1 | undetected | 0 | undetected | 0 | 0 | 0 | 0 | 1 | 2 |
| LAPC4 | **Orthotopically** | 10 | 12 | undetected | 5 | undetected | 1 | 11 | 3 | 2 | 8 | 42 |
|  | **Ectopically** | 8 | 2 | undetected | 0 | undetected | undetected | 3 | undetected | 2 | 2 | 9 |
| LAPC9 | **Orthotopically** | 7 | 6 | 7 | 3 | 5 | 3 | 12 | 4 | 7 | 14 | 61 |
|  | **Ectopically** | 10 | 3 | undetected | 1 | undetected | undetected | 2 | undetected | 1 | 1 | 8 |
