# Supplementary material for: Insights into homeobox B9: a propeller for metastasis in dormant prostate cancer progenitor cells
Source: Br J Cancer. 2021 Jul 10;125(7):1003–15. doi: 10.1038/s41416-021-01482-y (PMC8476533; doi:10.1038/s41416-021-01482-y)
Supplement: Supplementary file 9 — Email response about co-first author [file 41416_2021_1482_MOESM9_ESM.docx]

Dear Professor Tao Yang,

Thank you for your email and notifying us to the change you wish to make to the author list. In order to make the necessary change, please email your co-authors regarding the details of this change, and ask them to reply to your email confirming that they agree to these changes.  Once you have collected these replies, please combine all co-authors’ email responses in one document and upload this when submitting your final files.

Kind regards,

Reece Maggs

*(he/him/his -* [*more information why*](https://assets2.hrc.org/files/assets/resources/TalkingAboutPronouns_onesheet_FINAL.pdf?_ga=2.51103081.1903387523.1570435692-2144084816.1570435692)*)*

Editorial Assistant, Academic Journals

**Springer Nature**

4 Crinan Street London N1 9XW

T: +44 2078 432769

E: [reece.maggs@springernature.com](mailto:reece.maggs@springernature.com)

[nature.com](applewebdata://85DAA5A1-C2DB-4C20-B6D5-C59E3AEA72DD/www.nature.com) | [springernature.com](applewebdata://85DAA5A1-C2DB-4C20-B6D5-C59E3AEA72DD/www.springernature.com)

Reponses from all co-authors were as follows:

Responses from Qingtong Yi:

**Re:Add Dr Wei Zhang as co-first author**

| 发件人： | urologis... <urologis...@163.com> |
| --- | --- |
| 时   间： | 2021年05月28日 10:43:58 (星期五) |

| 收件人： | Yang Tao <yangt@fudan.edu.cn> |
| --- | --- |

Dr. Yang:

Thanks. I agree.

Sincerely,

QT Yi

At 2021-05-27 15:57:44, "Yang Tao" <yangt@fudan.edu.cn> wrote:

Dear all,

Thank everyone for your excellent work on this project "Insights into homeobox B9: A Propeller for Metastasis in Dormant Prostate Cancer Progenitor Cells".

As we all know that Dr. Wei Zhang did a great job and played an important role in experimental operation & data analysis during this project. But after several rounds of revision, Dr. Wei Zhang's labelling as co-first author was missing somehow from the manuscript. Now, we need to get everyone's permission to add Dr. Wei Zhang as a co-first author in addition to Dr. Yi Sui & Dr. Wei Hu.

If you agree, please response this email as soon as possible.

Thanks!

Regards,

Tao Yang

Responses from Qinghua You:

**Re:Add Dr Wei Zhang as co-first author**

| 发件人： | primatesh@126.com |
| --- | --- |
| 时   间： | 2021年05月28日 10:41:25 (星期五) |

| 收件人： | Yang Tao <yangt@fudan.edu.cn> |
| --- | --- |
| 抄　送： | suiyisy2005@sina.com  weihusurgeon@126.com  wzhang2000sjz@sina.com  dejiansh@yeah.net  hbzhu2000@126.com  urologyzrj@126.com  urologist_qtyi@163.com  tangtao@cuhk.edu.hk  upsetmonkey@163.com  mrszhushengjuan@163.com |

Dear Dr. Tao Yang:

I have no objection.

Dr. You Qinghua

At 2021-05-27 15:57:44, "Yang Tao" <[yangt@fudan.edu.cn](mailto:yangt@fudan.edu.cn)> wrote:

Dear all,

Thank everyone for your excellent work on this project "Insights into homeobox B9: A Propeller for Metastasis in Dormant Prostate Cancer Progenitor Cells".

As we all know that Dr. Wei Zhang did a great job and played an important role in experimental operation & data analysis during this project. But after several rounds of revision, Dr. Wei Zhang's labelling as co-first author was missing somehow from the manuscript. Now, we need to get everyone's permission to add Dr. Wei Zhang as a co-first author in addition to Dr. Yi Sui & Dr. Wei Hu.

If you agree, please response this email as soon as possible.

Thanks!

Regards,

Tao Yang

Responses from Rujian Zhu:

**Re:Add Dr Wei Zhang as co-first author**

| 发件人： | Dr. Ruji... <urologyzrj@126.com> |
| --- | --- |
| 时   间： | 2021年05月28日 10:38:18 (星期五) |

| 收件人： | Yang Tao <yangt@fudan.edu.cn> |
| --- | --- |
| 抄　送： | suiyisy2005@sina.com   weihusurgeon@126.com   wzhang2000sjz@sina.com   dejiansh@yeah.net   hbzhu2000@126.com   ...[[↓还有5个联系人]](javascript:void(0);) |

Dear Dr. Yang:

I agree.

RJ Zhu

At 2021-05-27 15:57:44, "Yang Tao" <[yangt@fudan.edu.cn](mailto:yangt@fudan.edu.cn)> wrote:

Dear all,

Thank everyone for your excellent work on this project "Insights into homeobox B9: A Propeller for Metastasis in Dormant Prostate Cancer Progenitor Cells".

As we all know that Dr. Wei Zhang did a great job and played an important role in experimental operation & data analysis during this project. But after several rounds of revision, Dr. Wei Zhang's labelling as co-first author was missing somehow from the manuscript. Now, we need to get everyone's permission to add Dr. Wei Zhang as a co-first author in addition to Dr. Yi Sui & Dr. Wei Hu.

If you agree, please response this email as soon as possible.

Thanks!

Regards,

Tao Yang

Responses from Shengjuan Zhu:

**Re:Add Dr Wei Zhang as co-first author**

| 发件人： | mrszhush... <mrszhush...@163.com> |
| --- | --- |
| 时   间： | 2021年05月28日 10:35:28 (星期五) |

| 收件人： | Yang Tao <yangt@fudan.edu.cn> |
| --- | --- |

Dear Prof. Yang:

Agree.

Zhu Shengjuan

At 2021-05-27 15:57:44, "Yang Tao" <[yangt@fudan.edu.cn](mailto:yangt@fudan.edu.cn)> wrote:

Dear all,

Thank everyone for your excellent work on this project "Insights into homeobox B9: A Propeller for Metastasis in Dormant Prostate Cancer Progenitor Cells".

As we all know that Dr. Wei Zhang did a great job and played an important role in experimental operation & data analysis during this project. But after several rounds of revision, Dr. Wei Zhang's labelling as co-first author was missing somehow from the manuscript. Now, we need to get everyone's permission to add Dr. Wei Zhang as a co-first author in addition to Dr. Yi Sui & Dr. Wei Hu.

If you agree, please response this email as soon as possible.

Thanks!

Regards,

Tao Yang

Reponses from Hongbo Zhu:

**Re:Add Dr Wei Zhang as co-first author**

| 发件人： | Dr. Hong... <hbzhu2000@126.com> |
| --- | --- |
| 时   间： | 2021年05月28日 10:33:00 (星期五) |

| 收件人： | Yang Tao <yangt@fudan.edu.cn> |
| --- | --- |
| 抄　送： | suiyisy2005@sina.com   weihusurgeon@126.com   wzhang2000sjz@sina.com   dejiansh@yeah.net   primatesh@126.com   ...[[↓还有5个联系人]](javascript:void(0);) |

Dr. Yang: I agree.

Sincerely,

Zhu Hongbo

At 2021-05-27 15:57:44, "Yang Tao" <[yangt@fudan.edu.cn](mailto:yangt@fudan.edu.cn)> wrote:

Dear all,

Thank everyone for your excellent work on this project "Insights into homeobox B9: A Propeller for Metastasis in Dormant Prostate Cancer Progenitor Cells".

As we all know that Dr. Wei Zhang did a great job and played an important role in experimental operation & data analysis during this project. But after several rounds of revision, Dr. Wei Zhang's labelling as co-first author was missing somehow from the manuscript. Now, we need to get everyone's permission to add Dr. Wei Zhang as a co-first author in addition to Dr. Yi Sui & Dr. Wei Hu.

If you agree, please response this email as soon as possible.

Thanks!

Regards,

Tao Yang

Responses from Lili Gao:

**Re:Add Dr Wei Zhang as co-first author**

| 发件人： | upsetmon... <upsetmon...@163.com> |
| --- | --- |
| 时   间： | 2021年05月28日 08:09:37 (星期五) |

| 收件人： | Yang Tao <yangt@fudan.edu.cn> |
| --- | --- |
| 抄　送： | suiyisy2005@sina.com  weihusurgeon@126.com  wzhang2000sjz@sina.com  dejiansh@yeah.net  hbzhu2000@126.com   ...[[↓还有5个联系人]](javascript:void(0);) |

Dear Prof. Yang:

Agree.

Yours,

Gao

At 2021-05-27 14:57:44, "Yang Tao" <[yangt@fudan.edu.cn](mailto:yangt@fudan.edu.cn)> wrote:

Dear all,

Thank everyone for your excellent work on this project "Insights into homeobox B9: A Propeller for Metastasis in Dormant Prostate Cancer Progenitor Cells".

As we all know that Dr. Wei Zhang did a great job and played an important role in experimental operation & data analysis during this project. But after several rounds of revision, Dr. Wei Zhang's labelling as co-first author was missing somehow from the manuscript. Now, we need to get everyone's permission to add Dr. Wei Zhang as a co-first author in addition to Dr. Yi Sui & Dr. Wei Hu.

If you agree, please response this email as soon as possible.

Thanks!

Regards,

Tao Yang

Responses from Dejian Li:

**Re:Add Dr Wei Zhang as co-first author**

| 发件人： | dejiansh <dejiansh@yeah.net> |
| --- | --- |
| 时   间： | 2021年05月27日 16:32:55 (星期四) |

| 收件人： | Yang Tao <yangt@fudan.edu.cn> |
| --- | --- |

Dear Dr. Yang:

Congrats! I agree.

Regards,

Li DJ

At 2021-05-27 15:57:44, "Yang Tao" <[yangt@fudan.edu.cn](mailto:yangt@fudan.edu.cn)> wrote:

Dear all,

Thank everyone for your excellent work on this project "Insights into homeobox B9: A Propeller for Metastasis in Dormant Prostate Cancer Progenitor Cells".

As we all know that Dr. Wei Zhang did a great job and played an important role in experimental operation & data analysis during this project. But after several rounds of revision, Dr. Wei Zhang's labelling as co-first author was missing somehow from the manuscript. Now, we need to get everyone's permission to add Dr. Wei Zhang as a co-first author in addition to Dr. Yi Sui & Dr. Wei Hu.

If you agree, please response this email as soon as possible.

Thanks!

Regards,

Tao Yang

Responses from Wei Zhang:

**回复：Add Dr Wei Zhang as co-first author**

| 发件人： | wzhang2000sjz@sina.com |
| --- | --- |
| 时   间： | 2021年05月27日 16:28:40 (星期四) |

| 收件人： | Yang Tao <yangt@fudan.edu.cn>  suiyisy2... <suiyisy2...@sina.com>  weihusur... <weihusur...@126.com>  dejiansh <dejiansh@yeah.net>   hbzhu2000 <hbzhu2000@126.com>   ...[[↓还有6个联系人]](javascript:void(0);) |
| --- | --- |

Dear Prof. Yang:

Thanks.

Regards,

W Zhang

----- 原始邮件 -----
发件人："Yang Tao" <[yangt@fudan.edu.cn](mailto:yangt@fudan.edu.cn)>
收件人：[suiyisy2005@sina.com](mailto:suiyisy2005@sina.com), [weihusurgeon@126.com](mailto:weihusurgeon@126.com), [wzhang2000sjz@sina.com](mailto:wzhang2000sjz@sina.com), [dejiansh@yeah.net](mailto:dejiansh@yeah.net), [hbzhu2000@126.com](mailto:hbzhu2000@126.com), [primatesh@126.com](mailto:primatesh@126.com), [urologyzrj@126.com](mailto:urologyzrj@126.com), [urologist_qtyi@163.com](mailto:urologist_qtyi@163.com), [tangtao@cuhk.edu.hk](mailto:tangtao@cuhk.edu.hk), [upsetmonkey@163.com](mailto:upsetmonkey@163.com), [mrszhushengjuan@163.com](mailto:mrszhushengjuan@163.com)
主题：Add Dr Wei Zhang as co-first author
日期：2021年05月27日 15点57分

Dear all,

Thank everyone for your excellent work on this project "Insights into homeobox B9: A Propeller for Metastasis in Dormant Prostate Cancer Progenitor Cells".

As we all know that Dr. Wei Zhang did a great job and played an important role in experimental operation & data analysis during this project. But after several rounds of revision, Dr. Wei Zhang's labelling as co-first author was missing somehow from the manuscript. Now, we need to get everyone's permission to add Dr. Wei Zhang as a co-first author in addition to Dr. Yi Sui & Dr. Wei Hu.

If you agree, please response this email as soon as possible.

Thanks!

Regards,

Tao Yang

Responses from Wei Hu:

**Re:Add Dr Wei Zhang as co-first author**

| 发件人： | weihusur... <weihusur...@126.com> |
| --- | --- |
| 时   间： | 2021年05月27日 16:23:08 (星期四) |

| 收件人： | Yang Tao <yangt@fudan.edu.cn> |
| --- | --- |
| 抄　送： | suiyisy2005@sina.com   wzhang2000sjz@sina.com   dejiansh@yeah.net   hbzhu2000@126.com   primatesh@126.com   ...[[↓还有5个联系人]](javascript:void(0);) |

Hello Dr. Yang:

Agree.

Dr. Wei Hu

Department of Urology

At 2021-05-27 15:57:44, "Yang Tao" <[yangt@fudan.edu.cn](mailto:yangt@fudan.edu.cn)> wrote:

Dear all,

Thank everyone for your excellent work on this project "Insights into homeobox B9: A Propeller for Metastasis in Dormant Prostate Cancer Progenitor Cells".

As we all know that Dr. Wei Zhang did a great job and played an important role in experimental operation & data analysis during this project. But after several rounds of revision, Dr. Wei Zhang's labelling as co-first author was missing somehow from the manuscript. Now, we need to get everyone's permission to add Dr. Wei Zhang as a co-first author in addition to Dr. Yi Sui & Dr. Wei Hu.

If you agree, please response this email as soon as possible.

Thanks!

Regards,

Tao Yang

Responses from Tao Tang:

**答复: Add Dr Wei Zhang as co-first author**

| 发件人： | Tao Tang... <tangtao@cuhk.edu.hk> |
| --- | --- |
| 时   间： | 2021年05月27日 16:15:43 (星期四) |

| 收件人： | Yang Tao <yangt@fudan.edu.cn>   suiyisy2... <suiyisy2...@sina.com>   weihusur... <weihusur...@126.com>   wzhang20... <wzhang20...@sina.com>   dejiansh... <dejiansh@yeah.net>   ...[[↓还有6个联系人]](javascript:void(0);) |
| --- | --- |

YES, AGREE.

Best Regrads

TAO

**发件人:** Yang Tao <[yangt@fudan.edu.cn](mailto:yangt@fudan.edu.cn)>
**发送时间:** 2021年5月27日 15:57
**收件人:** [suiyisy2005@sina.com](mailto:suiyisy2005@sina.com) <[suiyisy2005@sina.com](mailto:suiyisy2005@sina.com)>; [weihusurgeon@126.com](mailto:weihusurgeon@126.com) <[weihusurgeon@126.com](mailto:weihusurgeon@126.com)>; [wzhang2000sjz@sina.com](mailto:wzhang2000sjz@sina.com) <[wzhang2000sjz@sina.com](mailto:wzhang2000sjz@sina.com)>; [dejiansh@yeah.net](mailto:dejiansh@yeah.net) <[dejiansh@yeah.net](mailto:dejiansh@yeah.net)>; [hbzhu2000@126.com](mailto:hbzhu2000@126.com) <[hbzhu2000@126.com](mailto:hbzhu2000@126.com)>; [primatesh@126.com](mailto:primatesh@126.com) <[primatesh@126.com](mailto:primatesh@126.com)>; [urologyzrj@126.com](mailto:urologyzrj@126.com) <[urologyzrj@126.com](mailto:urologyzrj@126.com)>; [urologist_qtyi@163.com](mailto:urologist_qtyi@163.com) <[urologist_qtyi@163.com](mailto:urologist_qtyi@163.com)>; Tao Tang (OBG) <[tangtao@cuhk.edu.hk](mailto:tangtao@cuhk.edu.hk)>; [upsetmonkey@163.com](mailto:upsetmonkey@163.com) <[upsetmonkey@163.com](mailto:upsetmonkey@163.com)>; [mrszhushengjuan@163.com](mailto:mrszhushengjuan@163.com) <[mrszhushengjuan@163.com](mailto:mrszhushengjuan@163.com)>
**主题:** Add Dr Wei Zhang as co-first author

Dear all,

Thank everyone for your excellent work on this project "Insights into homeobox B9: A Propeller for Metastasis in Dormant Prostate Cancer Progenitor Cells".

As we all know that Dr. Wei Zhang did a great job and played an important role in experimental operation & data analysis during this project. But after several rounds of revision, Dr. Wei Zhang's labelling as co-first author was missing somehow from the manuscript. Now, we need to get everyone's permission to add Dr. Wei Zhang as a co-first author in addition to Dr. Yi Sui & Dr. Wei Hu.

If you agree, please response this email as soon as possible.

Thanks!

Regards,

Tao Yang

Responses from Yi Sui:

**回复：Add Dr Wei Zhang as co-first author**

| 发件人： | suiyisy2005@sina.com |
| --- | --- |
| 时   间： | 2021年05月27日 16:16:07 (星期四) |

| 收件人： | Yang Tao <yangt@fudan.edu.cn> |
| --- | --- |

Dear Prof. Yang:

I agree.

Yi Sui

----- 原始邮件 -----
发件人："Yang Tao" <[yangt@fudan.edu.cn](mailto:yangt@fudan.edu.cn)>
收件人：[suiyisy2005@sina.com](mailto:suiyisy2005@sina.com), [weihusurgeon@126.com](mailto:weihusurgeon@126.com), [wzhang2000sjz@sina.com](mailto:wzhang2000sjz@sina.com), [dejiansh@yeah.net](mailto:dejiansh@yeah.net), [hbzhu2000@126.com](mailto:hbzhu2000@126.com), [primatesh@126.com](mailto:primatesh@126.com), [urologyzrj@126.com](mailto:urologyzrj@126.com), [urologist_qtyi@163.com](mailto:urologist_qtyi@163.com), [tangtao@cuhk.edu.hk](mailto:tangtao@cuhk.edu.hk), [upsetmonkey@163.com](mailto:upsetmonkey@163.com), [mrszhushengjuan@163.com](mailto:mrszhushengjuan@163.com)
主题：Add Dr Wei Zhang as co-first author
日期：2021年05月27日 15点57分

Dear all,

Thank everyone for your excellent work on this project "Insights into homeobox B9: A Propeller for Metastasis in Dormant Prostate Cancer Progenitor Cells".

As we all know that Dr. Wei Zhang did a great job and played an important role in experimental operation & data analysis during this project. But after several rounds of revision, Dr. Wei Zhang's labelling as co-first author was missing somehow from the manuscript. Now, we need to get everyone's permission to add Dr. Wei Zhang as a co-first author in addition to Dr. Yi Sui & Dr. Wei Hu.

If you agree, please response this email as soon as possible.

Thanks!

Regards,

Tao Yang
